# Perioperative intravenous lidocaine for the prevention of chronic postsurgical pain: a systematic review and meta-analysis of randomized controlled trials

**DOI:** 10.3389/fmed.2026.1927770

**Published:** 2026-09-28

**Authors:** Wei-Jing Zhu, Ran An, Qian-Yun Pang, Xiao-Long Liang

**Affiliations:** 1 Inpatient Department, University Town Campus, Hospital of Chongqing University, Chongqing, China; 2 Department of Anesthesiology, Chongqing University Cancer Hospital, Chongqing, China

**Keywords:** chronic postsurgical pain, intravenous lidocaine, local anesthetic systemic toxicity, meta-analysis, neuropathic pain, perioperative analgesia, safety monitoring, systematic review

## Abstract

**Background:**

Chronic postsurgical pain (CPSP) poses a significant clinical burden, yet the efficacy of perioperative intravenous lidocaine for its prevention remains inconclusive. This meta-analysis evaluates its preventive efficacy and administration regimens.

**Methods:**

PubMed, EMBASE, and the Cochrane Library were searched from database inception to a prespecified cutoff of June 1, 2025; final electronic searches were run on June 8, 2025, to identify randomized controlled trials (RCTs) comparing perioperative intravenous lidocaine with placebo/standard care in adults. Primary outcomes were CPSP incidence at 3 and 6 months postoperatively.

**Results:**

Eighteen RCTs (1,391 patients) were included. Intravenous lidocaine significantly reduced CPSP incidence (RR = 0.67; 95% CI: 0.53 to 0.85; *P* = 0.001) and neuropathic pain risk (RR = 0.60; 95% CI: 0.43 to 0.85; *P* = 0.004) at 3 months. However, the reduction in the incidence of CPSP was not statistically significant at 6 months (RR = 0.68; 95% CI: 0.41 to 1.12; *P* = 0.13). Exploratory subgroup analyses of CPSP incidence at 3 months showed favorable pooled estimates in selected surgery and administration-regimen categories; however, they did not demonstrate a statistically significant difference between regimen categories. Safety assessment was limited by inconsistent adverse-event reporting and limited pharmacokinetic monitoring.

**Conclusion:**

Perioperative intravenous lidocaine reduces CPSP at 3 months, but evidence of an effect on CPSP incidence at 6 months remains inconclusive. Future trials should incorporate structured pharmacokinetic monitoring and exposure-response analyses and evaluate lidocaine's additive value alongside optimal regional anesthesia.

**Systematic Review Registration:**

PROSPERO, https://www.crd.york.ac.uk/PROSPERO/view/CRD420251063756, identifier CRD420251063756.

## 1 Introduction

Chronic postsurgical pain (CPSP) is defined as surgical pain that persists beyond three months and cannot be attributed to other conditions, including infection or malignancy recurrence (1). The incidence of CPSP varies across surgical procedures, and high-risk operations, including thoracotomy, radical mastectomy, and amputation, are associated with CPSP rates ranging from 10% to 50% (2–4). CPSP causes long-term patient suffering, including physical discomfort, anxiety, depression, and sleep disturbances (5–7), and is associated with increased risks of cardiovascular events and even dementia (8, 9). These consequences substantially contribute to both healthcare burdens and socioeconomic costs (10).

The pathogenesis of CPSP is complex and involves multiple factors, such as intraoperative nerve injury, peripheral and central sensitization, and persistent inflammatory responses (11–14). Consequently, the exploration of effective perioperative intervention strategies has become an important research topic in the fields of anesthesiology and pain medicine. Lidocaine, a classic amide local anesthetic, is commonly used in regional anesthesia and arrhythmia management. In recent years, its systemic administration during the perioperative period has gained increasing attention (15). Beyond its local anesthetic properties, intravenous lidocaine may attenuate the development of CPSP through systemic mechanisms, including the suppression of inflammatory mediators, blockade of nociceptive signaling, and modulation of dorsal horn neuronal activity (16–18).

However, current evidence from randomized controlled trials (RCTs) regarding the efficacy of intravenous lidocaine in preventing chronic postsurgical pain remains inconclusive. While some studies report significant reductions in the incidence and intensity of CPSP, others have observed no substantial benefits (19, 20). These discrepant findings may be attributed to methodological variations across studies, including differences in sample sizes, surgical procedures, lidocaine dosages, infusion rates, and administration duration.

Several meta-analyses have evaluated the effect of perioperative intravenous lidocaine on chronic postsurgical pain (21, 22) and have explored potential sources of heterogeneity. For example, Bailey et al. (21) examined dosing-related subgroups, whereas Hussain et al. (22) conducted subgroup analyses to investigate heterogeneity. Nevertheless, the limited number of eligible trials and substantial variation in surgical populations, lidocaine administration protocols, outcome definitions, and follow-up time points have precluded reliable conclusions regarding potential effect modifiers. An updated systematic evaluation incorporating more recent RCTs is therefore warranted. The primary aim of this study was to synthesize the latest RCT evidence regarding the efficacy of perioperative intravenous lidocaine for CPSP prevention. Furthermore, to address the aforementioned discrepancies, we aimed to investigate the observed clinical heterogeneity by examining specific clinical and administration-related parameters, including surgical type, continuous infusion rate, and administration period. Given the known heterogeneity in lidocaine trials, we anticipate that our exploratory findings will generate hypotheses regarding potentially relevant administration regimens, rather than provide definitive guidance, to better inform the perioperative use of intravenous lidocaine and direct future research.

## 2 Methods

### 2.1 Inclusion and exclusion criteria

Studies were selected on the basis of the following inclusion criteria: 1) studies that included adult patients (≥18 years) who underwent any surgical procedure, regardless of sex or ethnicity; 2) studies in which intravenous lidocaine was administered perioperatively; 3) studies in which a control group received placebo (e.g., normal saline) or standard care; 4) studies in which the incidence or intensity of chronic postsurgical pain (defined as pain persisting for ≥3 months postoperatively) was reported; 5) RCTs; and 6) studies for which the full text was available in English.

Studies were excluded if they 1) involved pediatric populations (<18 years); 2) were animal studies, reviews, case reports, or nonrandomized trials; 3) contained incomplete or duplicate data; or 4) utilized analgesic regimens that differed between the intervention and control groups (other than investigational lidocaine).

Specifically, studies permitting the use of perioperative nonsteroidal anti-inflammatory drugs (NSAIDs) or nerve blocks (including local infiltration) were eligible for inclusion, provided that the background analgesic protocol was identical for both the lidocaine and control groups.

### 2.2 Literature search strategy

This meta-analysis adhered to the Preferred Reporting Items for Systematic Reviews and Meta-Analyses (PRISMA) guidelines. The study protocol was prospectively registered with the PROSPERO international registry (Registration number: CRD 420251063756). To identify relevant studies, the PubMed, EMBASE, and Cochrane Library databases were systematically searched from their earliest records to June 1, 2025. The search strategy involved combining Medical Subject Headings (MeSH) with free-text terms to capture studies on lidocaine and chronic postoperative pain. Additionally, manual searches of the reference lists of the included studies were performed to identify additional relevant publications. The detailed search strategies for each database are provided in Appendix 1.

### 2.3 Changes from the registered protocol and review chronology

The protocol was first registered in PROSPERO on 31 May 2025 (Version 1.0). The final electronic database search was completed on 8 June 2025, and data extraction commenced on 10 October 2025. The PROSPERO record was updated to Version 2.0 on 16 November 2025 to add funding information; this update did not involve any changes to the review outcomes or methods. Version 3.0 was published on 18 November 2025.

The original protocol listed both chronic postsurgical pain and acute postoperative pain as primary outcomes. After data extraction had commenced, the review scope was refined to focus exclusively on chronic postsurgical pain. The decision to remove acute postoperative pain was made on 18 November 2025 and was recorded in PROSPERO Version 3.0 on the same day. This change was made because combining acute and chronic pain outcomes, which have distinct pathophysiological mechanisms, could obscure CPSP-specific findings. This was a *post hoc* scope amendment; it did not alter the study eligibility criteria, the extracted CPSP data, or the primary CPSP analyses. Acute postoperative pain outcome data were not included in the synthesis.

Although subgroup analysis was generally anticipated in the registered protocol, the specific subgroup variables—surgical type, lidocaine continuous infusion rate, and administration period—were selected *post hoc* to explore observed clinical heterogeneity. Similarly, the sensitivity analysis restricted to studies using exclusively intravenous administration was exploratory. These analyses should therefore be interpreted as hypothesis-generating.

### 2.4 Study selection and data extraction

#### 2.4.1 Study selection

Two investigators (Wei-Jing Zhu and Ran An) independently conducted the literature search and screened eligible randomized controlled trials (RCTs) by reviewing the titles, abstracts, and full texts. Data extraction was then performed independently using a standardized data extraction form. Any disagreements were resolved via discussion or consultation with a third investigator (Xiao-Long Liang).

#### 2.4.2 Data extraction and standardization protocol

Data extraction was performed independently using a standardized data extraction form. The following data were extracted: first author, publication year, type of surgery, sample size, patient baseline characteristics, and details of the lidocaine administration regimen, including the initial or single dose, continuous infusion rate, administration start and end time, and route of administration. To characterize and contextualize the potential variability in perioperative care, detailed components of the background analgesic protocols—including the use of peripheral nerve blocks, nonsteroidal anti-inflammatory drugs (NSAIDs), and other multimodal analgesia—were systematically extracted for tabulation and comparison between study groups. Safety data were derived from adverse events associated with lidocaine infusion.

To address the methodological heterogeneity inherent in CPSP assessment instruments and clinical constructs, we established an extraction hierarchy: (1) Incidence of CPSP. In studies reporting multiple pain thresholds, data reflecting “clinically meaningful pain” (defined as moderate-to-severe pain, e.g., NRS/VAS ≥ 4) were preferentially extracted over “any pain” to maximize clinical relevance. (2) Pain Intensity Constructs. For pain-intensity outcomes, one estimate per study was extracted at each follow-up time to avoid double counting. Resting pain was preferentially extracted when both resting and movement-evoked pain scores were available. Movement-evoked pain was extracted only when resting pain was not reported. Pain scores that were not explicitly distinguished by assessment condition, or were reported as a composite by the original investigators, were extracted as reported and were not classified as resting pain. We did not combine separately reported resting and movement-evoked pain scores within studies. The selected study-level estimates were pooled in the overall analysis of pain scores after harmonization to a common 0–10 scale. However, to address potential clinical heterogeneity driven by this pooling, we planned a specific sensitivity analysis prior to data synthesis. This analysis excluded studies that exclusively evaluated movement-evoked pain to confirm the reliability of the overall effect estimate. (3) Temporal Windows. For studies reporting follow-up across an aggregated timeframe (e.g., “3–6 months”), a predefined approximation rule was applied, assigning the data to the nearest follow-up endpoint (e.g., 3 months) to prevent unnecessary study exclusion.

Pain intensity scores were obtained using the numerical rating scale (NRS), the visual analog scale (VAS), the verbal rating scale (VRS), and the pain severity items of the Brief Pain Inventory (BPI). To allow statistical pooling and direct clinical interpretation, all scores were systematically harmonized to a common 0–10 scale. Specifically, the BPI pain severity items and standard NRS/VRS are intrinsically measured on a 0–10 scale, while any VAS scores originally reported on a 0–100 mm scale were converted by dividing the values by 10. For data presented only in graphical format, numerical values were extracted using WebPlotDigitizer (23). For continuous outcomes, the mean and standard deviation (SD) values were extracted directly. When studies reported data as medians and interquartile ranges (IQRs), the data were converted to means ± SDs using the methods described by Wan et al. and Luo et al. (24, 25).

A study by Toner et al. combined postoperative subcutaneous infusion with systemic intravenous lidocaine (26). While sharing a broad pharmacological rationale, subcutaneous infusion is not pharmacokinetically equivalent to strictly intravenous administration. This study was retained for the primary analysis, with its impact further evaluated via sensitivity analysis. For Hojski et al. (27), persistent pain at 90 and 180 days was assessed during coughing. Group-specific event counts were reconstructed from the individual graphical data at these follow-up visits, with any reported pain during coughing classified as persistent postsurgical pain. The reconstructed totals matched the aggregate numbers reported in the original article: 9 of 52 patients at 90 days and 4 of 52 patients at 180 days. A *post hoc* descriptive synthesis was undertaken for studies reporting movement-evoked pain scores at approximately 3 months after surgery. For each study, the mean difference was calculated as the lidocaine-group mean minus the control-group mean, with 95% confidence intervals calculated using Welch's unequal-variance two-sample *t* method. No pooled estimate was calculated because the pain-evoking conditions and data sources differed across studies.

### 2.5 Risk of bias assessment and certainty of evidence

Two reviewers (Ran An and Qian-Yun Pang) independently assessed the risk of bias for the included randomized trials using the revised Cochrane Risk of Bias tool (RoB 2) (28). The assessment encompassed the following five domains: 1) bias arising from the randomization process; 2) bias due to deviations from intended interventions; 3) bias due to missing outcome data; 4) bias in measurements of the outcome; and 5) bias in the selection of the reported result. On the basis of these domains, the overall risk of bias for each study was categorized as “low risk,” “some concerns,” or “high risk.” Any disagreements were resolved via consensus or consultation with a third reviewer (Xiao-Long Liang).

Following the risk of bias assessment, the certainty of evidence for each assessed outcome was evaluated using the Grading of Recommendations, Assessment, Development, and Evaluation (GRADE) approach (29). Evidence was graded as high, moderate, low, or very low certainty on the basis of the assessment of five domains: risk of bias, inconsistency, indirectness, imprecision, and publication bias.

### 2.6 Data analysis

Statistical analyses were performed using Review Manager (version 5.4; The Cochrane Collaboration) and Stata (version 17; StataCorp). The *post hoc* REML-HKSJ sensitivity analyses were conducted in R (version 4.6.1; meta package version 8.5-0).

For dichotomous outcomes (e.g., incidence of CPSP), the relative risk (RR) with 95% confidence interval (CI) was calculated. For continuous outcomes, the choice of effect measure depended on the feasibility of scale harmonization. Because postoperative pain intensity scores could be successfully harmonized to a common 0–10 scale, the mean difference (MD) with 95% CI was calculated. Conversely, for other continuous outcomes where direct scale harmonization was not feasible across diverse instruments (e.g., total SF-MPQ scores), the standardized mean difference (SMD) with 95% CI was utilized. Heterogeneity was assessed using the *I*^2^ statistic. An *I*^2^ value >50% was considered indicative of substantial heterogeneity. Given the anticipated clinical and methodological diversity (e.g., varying surgical types and infusion protocols), a random-effects model was applied *a priori* for all analyses to provide a conservative estimate of the pooled effect size. Specifically, the DerSimonian and Laird (DL) method was employed to estimate between-study variance. As a *post hoc* robustness check, the two primary CPSP outcomes were reanalyzed in R using restricted maximum likelihood estimation of between-study variance combined with the Hartung–Knapp–Sidik–Jonkman adjustment for confidence intervals.

Subgroup analyses were used to explore potential sources of clinical and methodological heterogeneity. Meta-regression was performed only when at least 10 studies were available. When fewer than 10 studies were available, any subgroup analyses were considered exploratory and interpreted cautiously. Subgroup strata containing a single study were presented descriptively and were not interpreted as providing evidence of a subgroup-specific treatment effect. Although the general use of subgroup analyses to address heterogeneity was planned in our PROSPERO protocol, the specific categorical variables were determined *post hoc* as exploratory analyses to investigate clinical variations observed during data extraction. These exploratory subgroups were categorized on the basis of clinical rationale (30–32). Surgery type was categorized as breast surgery vs. non-breast surgery. Breast surgery was chosen as a separate category because it contributed a relatively large number of cases and had relatively homogeneous clinical characteristics. This dichotomy was not intended to imply clinical equivalence among the non-breast procedures, which may differ in the pattern of surgical tissue and nerve injury as well as in baseline CPSP risk. Administration period was categorized as intraoperative only vs. extended postoperative administration, because the timing of lidocaine exposure may influence perioperative nociceptive input and central sensitization. Continuous infusion rate was categorized as <2 vs. ≥2 mg/kg/h because these thresholds reflected the distribution of the reported continuous infusion regimens and provided clinically interpretable groups for exploratory comparison. These analyses were intended to explore potential sources of between-study heterogeneity and were not designed to establish an optimal regimen. Finally, to evaluate the robustness of our findings and the impact of methodological variances, sensitivity analyses were conducted by: 1) Employing the leave-one-out method; 2) Excluding Hojski et al. (27), for which only cough-evoked pain scores were available for extraction at 3 months, to assess the influence of including this assessment condition on the pooled pain-intensity estimate; and 3) Excluding the study utilizing a mixed intervention protocol [Toner et al., IV + subcutaneous infusion (26)], to assess the influence of this non-intravenous cointervention on the pooled estimates for CPSP incidence at 3 and 6 months.

## 3 Results

### 3.1 Literature screening process and results

The initial database search identified a total of 3,618 records. After 702 duplicates were removed, 2,916 records were screened by title and abstract, of which 2,870 were excluded as irrelevant. A total of 46 full-text articles were subsequently assessed for eligibility. Following the full-text review, 18 randomized controlled trials involving 1,391 patients met the inclusion criteria for this systematic review (19, 20, 26, 27, 33–46). The detailed study selection process is summarized in a PRISMA flow diagram (Figure 1).

**Figure 1:**
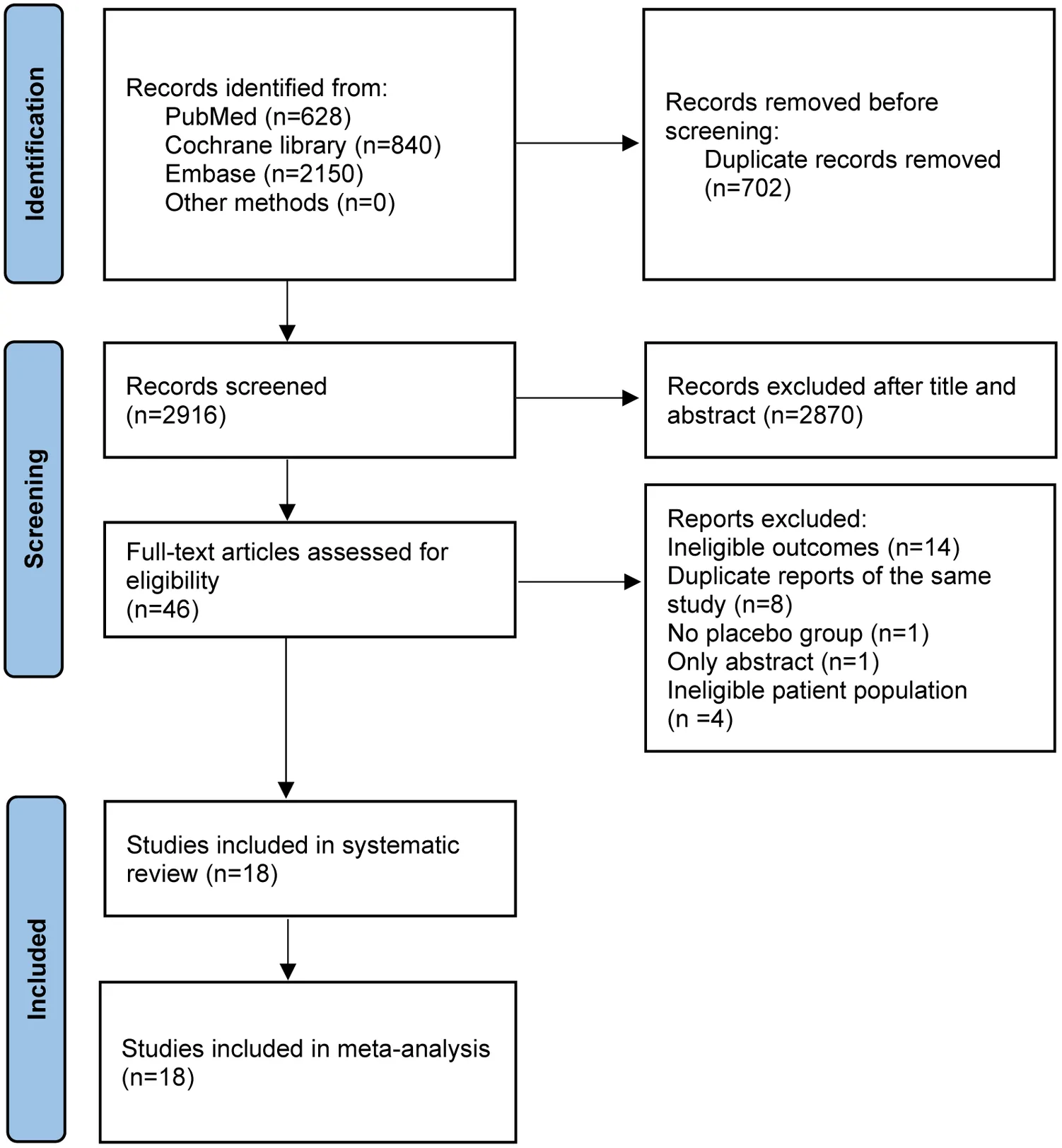
PRISMA flow diagram of study selection.

### 3.2 Characteristics of the included studies and risk of bias assessment

The detailed characteristics of the 18 included RCTs are summarized in Table 1. The included trials were published between 2012 and 2024, with sample sizes ranging from 36 to 150 participants. The types of surgery varied and included breast surgery, thoracic surgery, abdominal procedures, spine fusion, head and neck cancer surgery, and robotic thyroidectomy. The lidocaine administration protocols generally involved an intravenous bolus (1.5–3 mg/kg) followed by continuous infusion, with rates ranging from 1 to 4 mg/kg/h and durations of up to 48 h. However, a detailed evaluation of the pharmacokinetic parameters revealed substantial methodological heterogeneity across the included trials (Supplementary Table 1).

**Table 1:** Characteristics of included randomized controlled trials.

Study	ASA	Sample size (L/P)	Surgical procedure	Lidocaine initial/single dose	Lidocaine continuous infusion rate	Administration start	End of lidocaine administration	Control group	Outcomes assessed	Notes
Grigoras et al. (33)	I–II	17/19	Breast Surgery	1.5 mg/kg	1.5 mg/kg/h	After intubation	1 h postoperatively	Saline	①③④	
Terkawi et al. (46)	I–III	34/27	Breast Surgery	1.5 mg/kg	2 mg/kg/h	At induction	2 h after PACU arrival/discharge	Saline	①	
Choi et al. (34)	I–II	41/43	Robotic Thyroidectomy	2 mg/kg	3 mg/kg/h	At induction	At extubation	Saline	①③④	^‡^
Kim et al. (35)	I–II	39/39	Breast Surgery	2 mg/kg	2 mg/kg/h	At induction	Before transfer to recovery	Saline	①④	
Jendoubi et al. (36)	I–II	20/20	Open Nephrectomy	1.5 mg/kg	1 mg/kg/h	At induction	24 h postoperatively	Saline	②	
Beaussier et al. (20)	I–III	29/27	Laparoscopic Colorectal	60 mg (6 mL of 1%)	60 mg/h (4 mL/h of 1.5%)	Postoperatively	48 h postoperatively	Saline	①②	
Kendall et al. (37)	I–III	74/74	Breast Surgery	1.5 mg/kg	2 mg/kg/h	At induction	1 h postoperatively	Saline	①③④	
Ibrahim et al. (38)	I–III	20/20	Spinal Fusion	2 mg/kg	3 mg/kg/h	At induction	End of surgery	Saline	③	
Khan et al. (39)	NS	51/49	Breast Surgery	1.5 mg/kg	2 mg/kg/h	At induction	At surgical suture	Saline	①②③④	^§^
Ghimire et al. (19)	I–II	32/32	Laparoscopic Inguinal Hernia	1.5 mg/kg	2 mg/kg/h	At induction	At extubation	Saline	①	
Dai et al. (40)	I–II	58/57	Gastrointestinal Tumor	1.5 mg/kg	1.5 mg/kg/h	After intubation	At surgical suture	Saline	①③	
Toner et al. (26)	I–III	74/76	Breast Surgery	1.5 mg/kg	2 mg/kg/h	At induction	12 h postoperatively	Saline	①	^#^
Xia et al. (41)	I–III	41/41	Breast Surgery	1.5 mg/kg	2 mg/kg/h	At induction	End of surgery	Saline	①②③④	
Lu et al. (42)	II–III	29/28	Thoracoscopic Pneumonectomy	1.5 mg/kg	2 mg/kg/h (intra-op) + PCA	At induction	Intra-op + 48 h PCA	Saline	①③	^¶^
Rekatsina et al. (44)	I–II	25/25	Open Abdominal Gynecological	1.5 mg/kg	1.5 mg/kg/h	At induction	End of surgery	Saline	②③	
Wallon et al. (43)	NS	57/61	Head & Neck Cancer Surgery	1.5 mg/kg	2→1 mg/kg/h (PACU)	Before skin incision	At PACU discharge	5% Glucose	①③④	*
Abdel Rady et al. (45)	I–II	30/30	Spine Fusion	4 mg/kg in 50 mL over 30 min	None	At induction	30 min after initiation	Saline	③	^♪^
Hojski et al. (27)	I–III	28/24	Thoracoscopic Surgery	1.5 mg/kg	3 mg/kg/h	30 min before incision	2 h postoperatively	Saline	①③	^†^

L, lidocaine group; P, Placebo group; NS, Not specified; PCA, patient-controlled analgesia; PACU, post-anesthesia care unit; CPSP, Chronic Postsurgical Pain.

① The incidence of CPSP.

② The incidence of Neuropathic pain at 3 months.

③ The pain scores at 3 months.

④ The SF-MPQ scores at 3 months.

* Data reported for combined “3–6 months” time point.

† This study only reported pain scores during coughing.

‡ Outcomes ③ and ④ calculated for CPSP patients only.

§ Outcome ④ assessed using SF-MPQ-2.

¶ Outcome ③ calculated for CPSP patients only.

# Intravenous infusion stopped at end of surgery, followed by subcutaneous lidocaine infusion on ward for up to 12 h.

♪ Single 30 min lidocaine infusion without a subsequent continuous infusion.

Specifically, pharmacokinetic protocols lacked standardization, particularly regarding weight-scaling metrics. Critically, with the exception of Hojski and Toner et al. (26, 27), who clearly reported dosing based on actual body weight, the majority of the included trials did not explicitly state whether lidocaine doses were calculated based on ideal body weight (IBW) or actual body weight; instead, these trials reported unadjusted weight-based dosing (e.g., mg/kg). This omission is significant, particularly for obese patients, where using actual body weight can lead to accidental overdose. Furthermore, the rate of bolus administration varied considerably, ranging from rapid unspecified pushes to slow infusions over 10 to 30 min. Some trials even utilized a fixed dose for postoperative maintenance regardless of weight [e.g., Beaussier et al. (20)]. Notably, the majority of the included studies did not perform routine plasma lidocaine level monitoring, and criteria for dose adjustment or discontinuation in response to early toxicity signs were frequently omitted or poorly defined.

The risk of bias assessment results are presented in Figure 2. Overall, the majority of studies were assessed as having a low risk of bias, while several raised some concerns. Figure 2 provides a descriptive study-level summary of the domain-based RoB 2 assessments. In the GRADE assessment, risk-of-bias considerations were evaluated in relation to each outcome, with particular attention to the completeness of long-term follow-up and the potential influence of missing outcome data (Supplementary Table 3).

**Figure 2:**
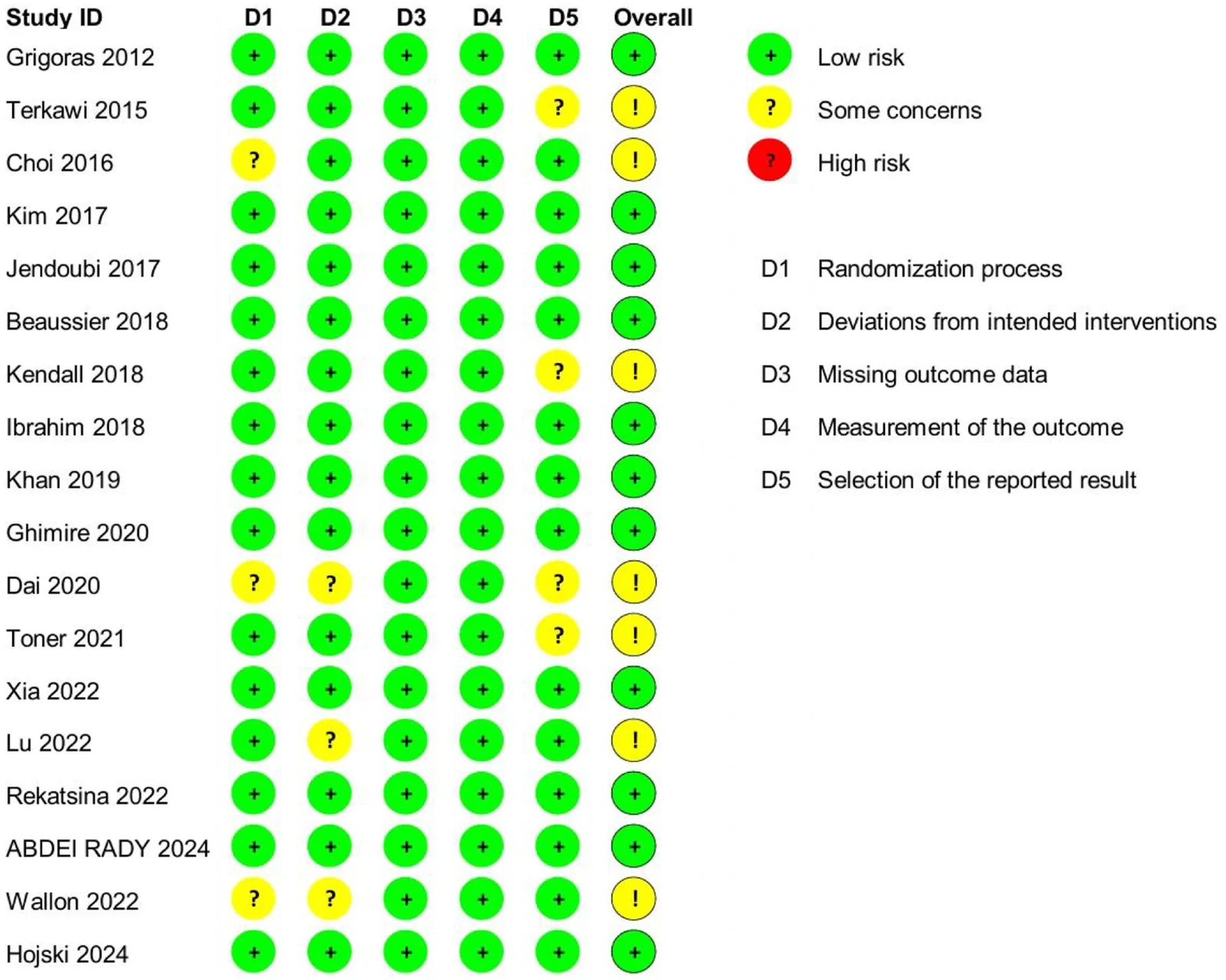
Risk of bias assessment for the included randomized controlled trials.

### 3.3 Meta-analysis results

#### 3.3.1 Primary outcomes

##### 3.3.1.1 Incidence of chronic postsurgical pain at 3 and 6 months

Thirteen RCTs (19, 20, 26, 27, 33–35, 37, 39–43) (*n* = 1,029) evaluated the incidence of CPSP at 3 months postsurgery (Figure 3A). Meta-analysis revealed that compared with the control, intravenous lidocaine infusion significantly reduced the risk of CPSP (RR = 0.67; 95% CI: 0.53 to 0.85; *P* = 0.001), with low heterogeneity observed (*I*^2^ = 19%).

**Figure 3:**
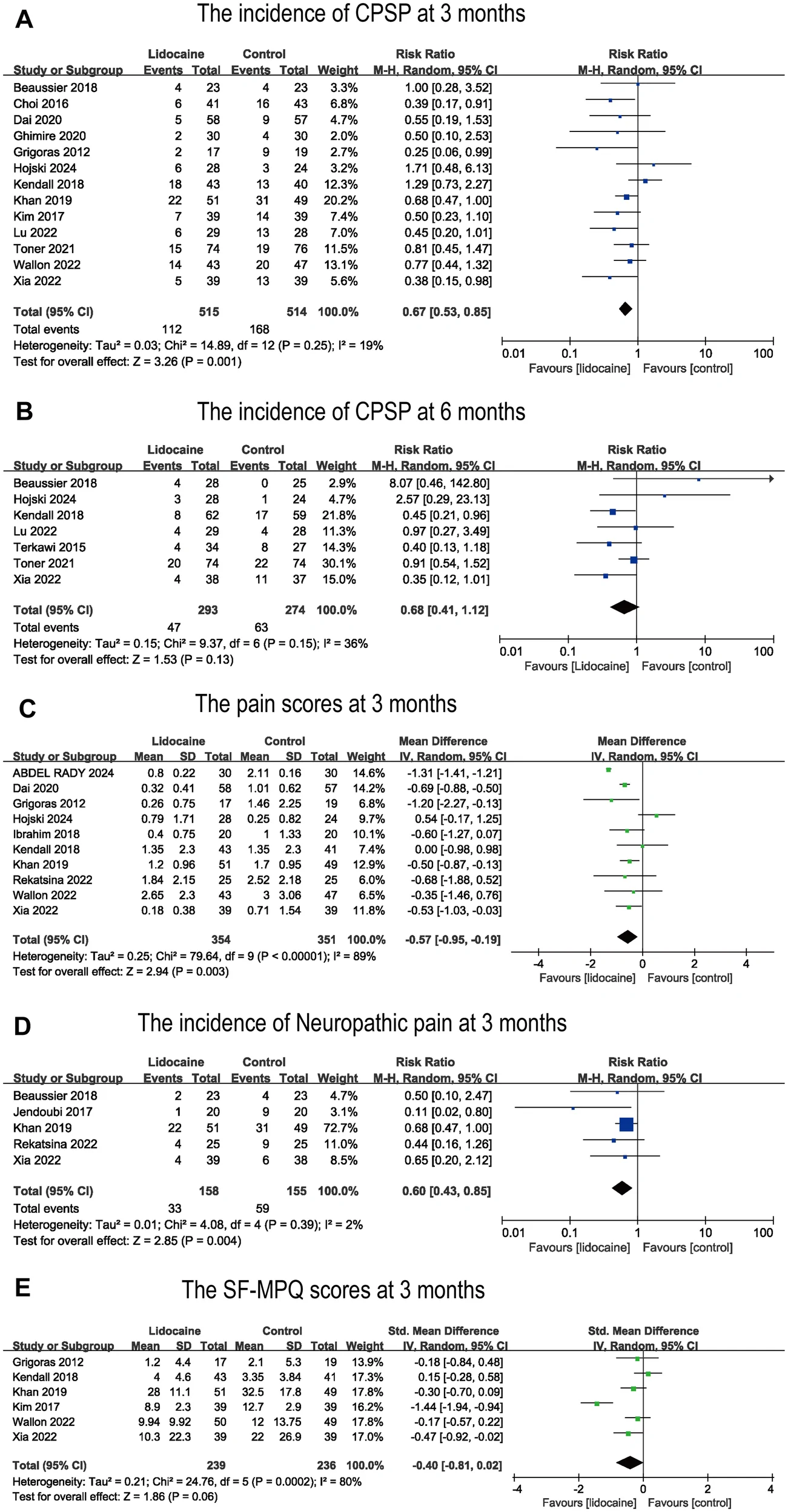
Pooled effects of perioperative intravenous lidocaine on chronic postsurgical pain and secondary pain outcomes. **(A)** CPSP incidence at 3 months. **(B)** CPSP incidence at 6 months. **(C)** Pain scores at 3 months. **(D)** Neuropathic pain incidence at 3 months. **(E)** SF-MPQ scores at 3 months. CPSP, chronic postsurgical pain; SF-MPQ, Short-Form McGill Pain Questionnaire.

According to the subgroup analyses (Figure 4), a significant reduction in risk was consistently observed for both breast surgery (RR = 0.68; 95% CI: 0.47 to 0.98; *P* = 0.04; *I*^2^ = 45%) and other surgical types (RR = 0.64; 95% CI: 0.46 to 0.90; *P* = 0.009; *I*^2^ = 0%). With respect to administration protocols, subgroup analyses showed statistically significant pooled estimates within several surgery-type and administration-regimen categories. However, meta-regression analysis did not identify surgery type, continuous infusion rate, or administration period as statistically significant effect modifiers (all *P* > 0.05). These exploratory analyses therefore did not establish a superior administration regimen.

**Figure 4:**
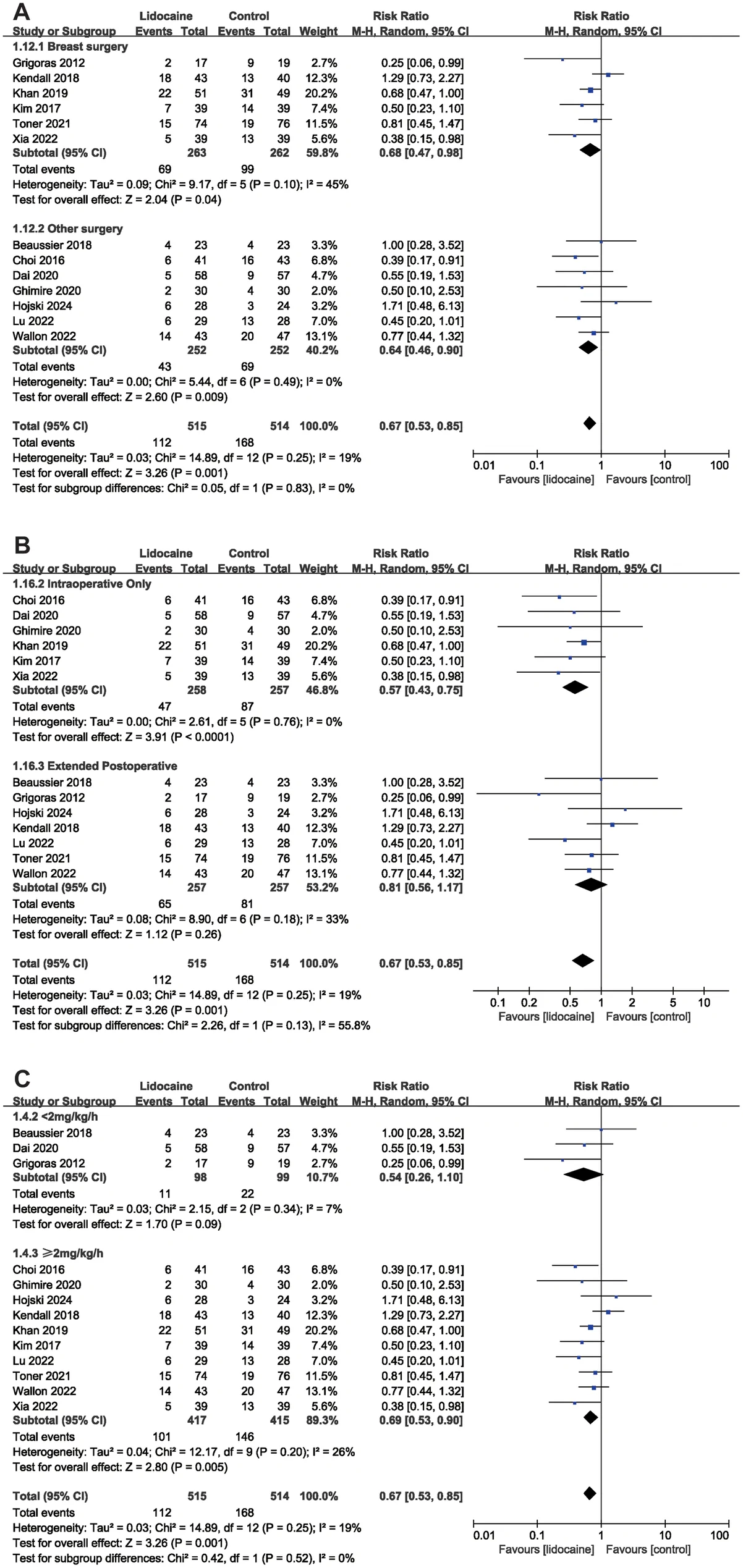
Subgroup analyses of CPSP incidence at 3 months according to **(A)** surgery type (breast versus other surgery), **(B)** administration period (intraoperative only versus extended postoperative administration), and **(C)** continuous infusion rate (<2 versus ≥2 mg/kg/h). CPSP, chronic postsurgical pain.

However, at 6 months, the pooled estimate was imprecise and did not show a statistically significant reduction in CPSP incidence. An analysis of the incidence of CPSP at 6 months (Figure 3B), which included seven RCTs (20, 26, 27, 37, 41, 42, 46) (*n* = 567), indicated that the overall effect of lidocaine was not statistically significant (RR = 0.68; 95% CI: 0.41 to 1.12; *P* = 0.13; *I*^2^ = 36%).

In exploratory *post hoc* subgroup analyses (Figure 5), the pooled estimates within the breast-surgery subgroup (RR = 0.56; 95% CI: 0.34 to 0.92; *P* = 0.02; *I*^2^ = 35%), and the ≥2 mg/kg/h continuous infusion rate subgroup (RR = 0.64; 95% CI: 0.41 to 0.99; *P* = 0.04) favored lidocaine. However, the tests for subgroup differences were not statistically significant (*P* = 0.08 and *P* = 0.09, respectively). Given that only seven studies were available, these findings are hypothesis-generating and do not establish a superior surgical population or continuous infusion regimen.

**Figure 5:**
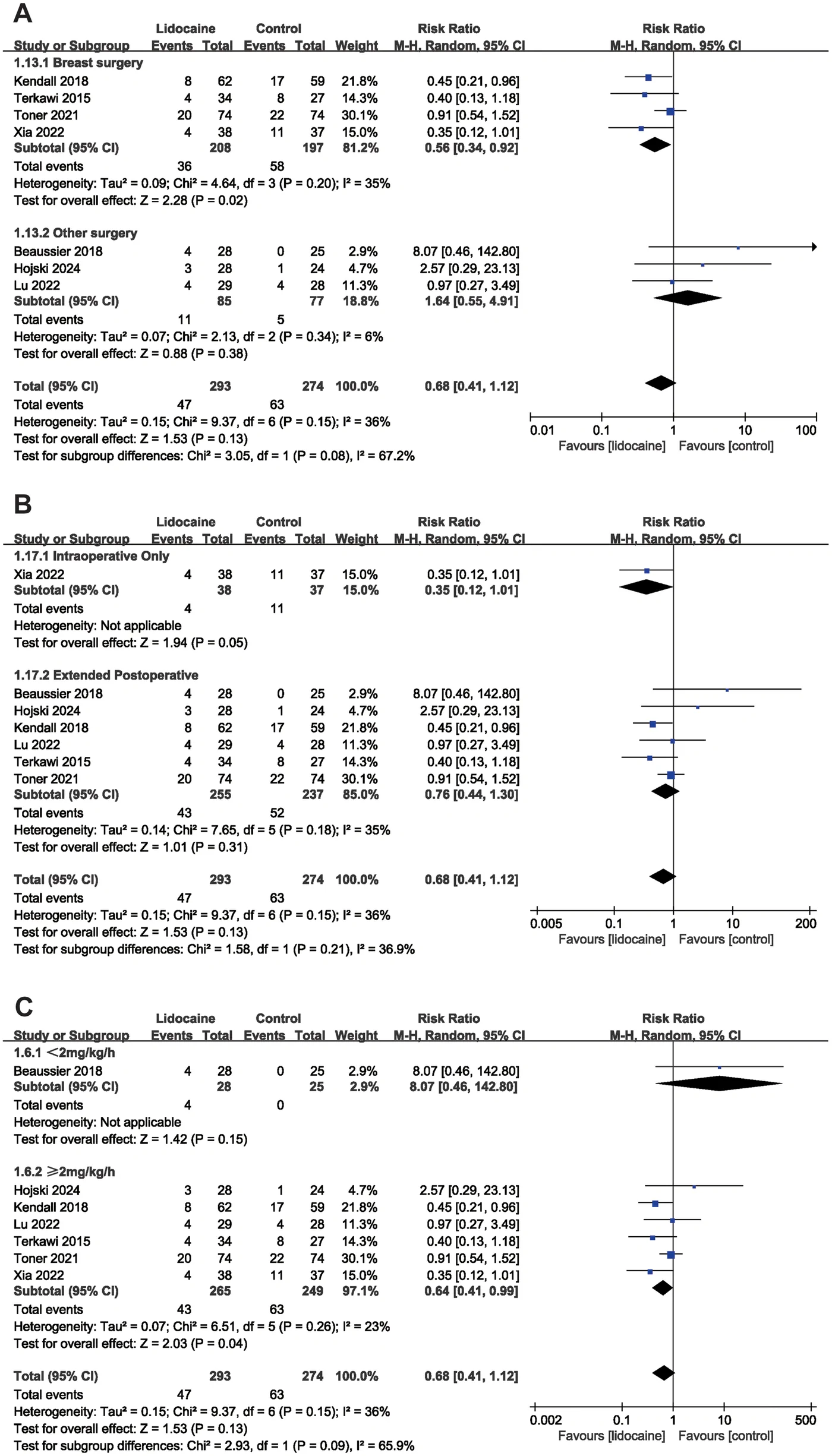
Subgroup analyses of CPSP incidence at 6 months according to **(A)** surgery type (breast versus other surgery), **(B)** administration period (intraoperative only versus extended postoperative administration), and **(C)** continuous infusion rate (<2 versus ≥2 mg/kg/h).

In summary, perioperative intravenous lidocaine was associated with a lower CPSP incidence at 3 months, whereas the evidence at 6 months remained inconclusive; no surgery-specific or regimen-specific long-term benefit can be established.

#### 3.3.2 Secondary outcomes

##### 3.3.2.1 Pain scores at 3 months after surgery

Ten studies (27, 33, 37–41, 43–45) reported pain scores at 3 months postsurgery (Figure 3C). Pooled analysis revealed that patients in the lidocaine group had significantly lower pain scores than those in the control group did (MD = −0.57; 95% CI: −0.95 to −0.19; *P* = 0.003). However, substantial heterogeneity was observed (*I*^2^ = 89%).

Exploratory meta-regression analyses did not identify surgery type (*P* = 0.838) or administration period (*P* = 0.129) as statistically significant effect modifiers. Similarly, subgroup analyses according to surgery type and administration period did not demonstrate statistically significant between-subgroup differences (*P* = 0.81 and *P* = 0.19, respectively). Abdel Rady et al. (45) used a single 30 min lidocaine infusion without subsequent continuous infusion and was therefore not included in the subgroup analysis according to continuous infusion rate. Among the remaining nine studies with continuous lidocaine infusion, the pooled estimate was more favorable in the <2 mg/kg/h subgroup (MD = −0.71, 95% CI: −0.89 to −0.52; *I*^2^ = 0%) than in the ≥2 mg/kg/h subgroup (MD = −0.31, 95% CI: −0.65 to 0.03; *I*^2^ = 38%). The test for subgroup differences was statistically significant (*P* = 0.04) (Figure 6C). Because this analysis was *post hoc*, involved only nine studies, and was one of multiple exploratory subgroup comparisons, it should be interpreted as hypothesis-generating and not as evidence that a lower continuous infusion rate is superior or optimal. When stratified by administration period, heterogeneity declined in the Extended Postoperative subgroup (*I*^2^ = 59%), but remained high in the intraoperative-only subgroup (*I*^2^ = 90%). No significant difference was observed between the administration period subgroups (*P* = 0.19). When stratified by surgery type, heterogeneity was eliminated within the breast surgery subgroup (*I*^2^ = 0%), but remained high in the “other surgery” subgroup (*I*^2^ = 92%). No significant difference was observed between the surgery type subgroups (*P* = 0.81).

**Figure 6:**
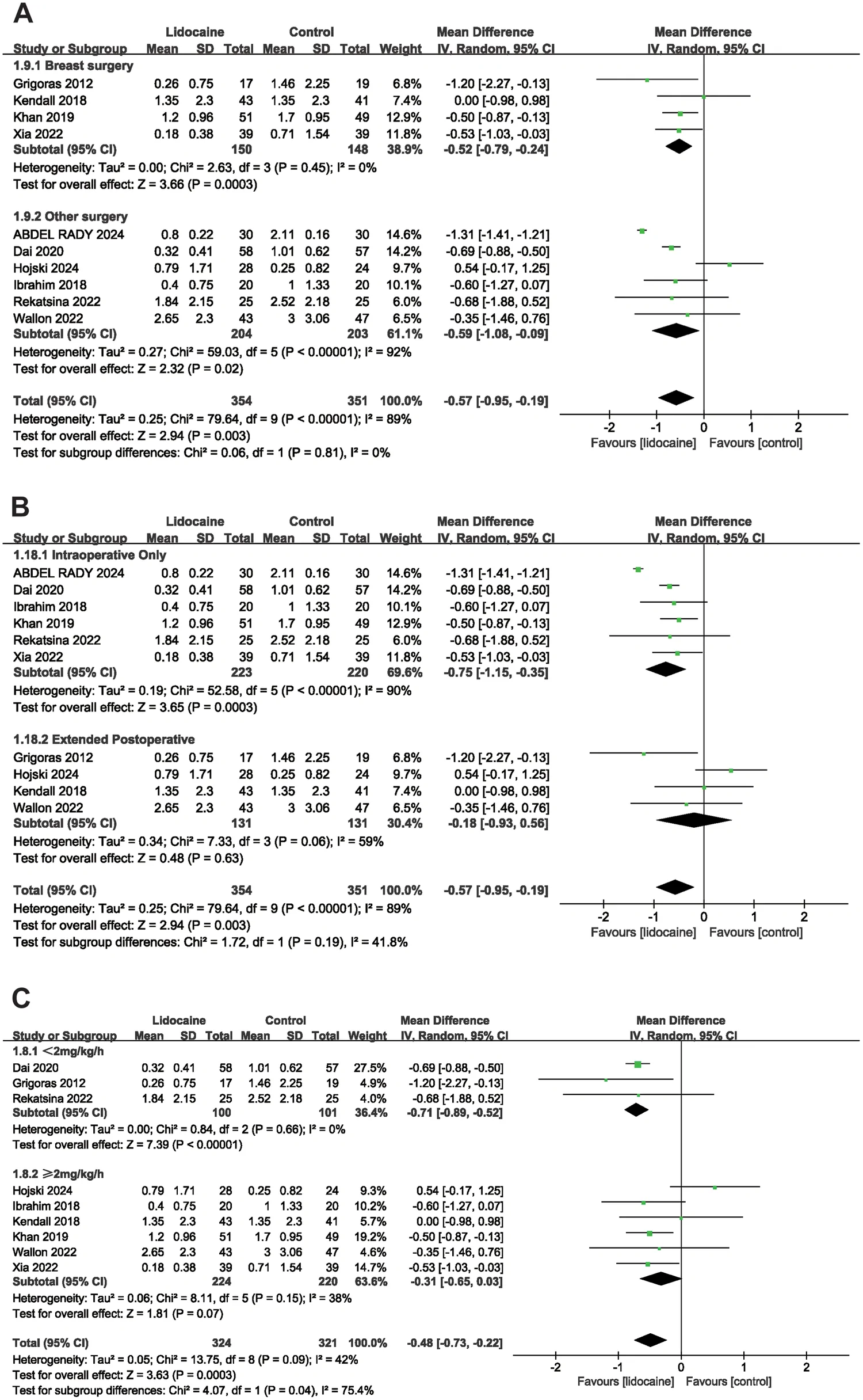
Subgroup analysis of pain scores at 3 months by surgery type **(A)**, administration period **(B)**, and continuous infusion rate **(C)**. Abdel Rady et al. was excluded from panel C because lidocaine was administered as a single 30 min infusion without a subsequent continuous infusion.

Three studies reported continuous movement-evoked pain scores at approximately 3 months after surgery (Supplementary Table 5). Pain was assessed during ipsilateral arm abduction (39), unspecified movement (45), or coughing, respectively (27). Abdel Rady et al. (45) reported lower movement-evoked pain in the lidocaine group (MD: −1.00, 95% CI: −1.21 to −0.79), whereas Khan et al. (39) showed no clear between-group difference (MD: −0.40, 95% CI: −0.94 to 0.14). In Hojski et al. (27), the graphically reconstructed estimate for cough-evoked pain was imprecise and numerically favored the control group (MD: 0.54, 95% CI: −0.20 to 1.28). Given the differences in pain-evoking maneuvers and the graphical derivation of outcome data, these results were not quantitatively pooled.

##### 3.3.2.2 Incidence of neuropathic pain and SF-MPQ scores

With respect to neuropathic pain, a meta-analysis of five studies (20, 36, 39, 41, 44) revealed that lidocaine significantly reduced the risk at 3 months after surgery (RR = 0.60; 95% CI: 0.43 to 0.85; *P* = 0.004; *I*^2^ = 2%) (Figure 3D), with minimal heterogeneity observed.

In contrast, the pooled results from six studies (33, 35, 37, 39, 41, 43) reporting Short-Form McGill Pain Questionnaire (SF-MPQ) scores were not significantly different between the two groups (SMD = −0.40; 95% CI: −0.81 to 0.02; *P* = 0.06; *I*^2^ = 80%) (Figure 3E). Owing to the limited number of studies included, the sources of the substantial heterogeneity observed for this outcome could not be further explored.

### 3.4 Sensitivity analysis, publication bias, and GRADE assessment

Sensitivity analyses described in the Methods were conducted. First, the leave-one-out analysis demonstrated the robustness of the results for the incidence of CPSP at 3 months, pain scores at 3 months, and the incidence of neuropathic pain at 3 months. Conversely, the results for the incidence of CPSP at 6 months and SF-MPQ scores at 3 months demonstrated sensitivity to the exclusion of individual studies (Supplementary Figures 1–5). Specifically, regarding the incidence of CPSP at 6 months, excluding the study by Beaussier et al. (20) shifted the pooled estimate to a significant result (RR = 0.64; 95% CI: 0.41 to 0.98; *P* = 0.043; *I*^2^ = 23%). For SF-MPQ scores at 3 months, excluding the study by Kendall et al. resulted in a significant pooled effect size (SMD = −0.51; 95% CI: −0.95 to −0.07, *P* = 0.024, *I*^2^ = 78%). We also conducted a HKSJ sensitivity analysis on the incidence of chronic pain at 3 months and 6 months after the surgery (Supplementary Figures 11, 12). For CPSP incidence at 3 months, the REML-HKSJ random-effects model showed a significant reduction with perioperative intravenous lidocaine (RR = 0.68; 95% CI: 0.52 to 0.88; *P* = 0.007; *I*^2^ = 18.8%). For CPSP incidence at 6 months, the effect remained inconclusive (RR = 0.67; 95% CI: 0.36 to 1.24; *P* = 0.166; *I*^2^ = 35.8%).

Furthermore, sensitivity analysis was performed by excluding the single study that exclusively evaluated movement-evoked pain [Hojski et al. (27)]. This exclusion did not materially alter the pooled effect estimates for postoperative pain scores at 3 months (MD = −0.70; 95% CI: −1.06 to −0.34; *P* = 0.0001; *I*^2^ = 87%). Excluding the study using a mixed intravenous and postoperative subcutaneous lidocaine protocol [Toner et al. (26)] did not change the overall interpretation of the CPSP outcomes. At 3 months, the pooled effect remained statistically significant (RR = 0.65; 95% CI: 0.50 to 0.85; *P* = 0.002; *I*^2^ = 24%). At 6 months, the estimate remained imprecise and was not statistically significant (RR = 0.60; 95% CI: 0.32 to 1.12; *P* = 0.11; *I*^2^ = 31%). In addition to the prespecified analyses, two exploratory *post hoc* sensitivity analyses were performed regarding postoperative pain scores at 3 months to further investigate observed heterogeneity. First, to mitigate potential bias where Wan's conversion method (24, 25) might underestimate variance, studies utilizing this method [Kendall and Khan (37, 39)] were excluded. The results remained robust (MD = −0.63; 95% CI: −1.06 to −0.21, *P* = 0.003), although heterogeneity persisted (*I*^2^ = 89%). Second, the study by Abdel Rady et al. (45) was identified as an extreme outlier, reporting an implausibly large effect size (MD = −1.31). Following its exclusion, the pooled effect size was adjusted to MD = −0.48 (95% CI: −0.73 to −0.22; *P* = 0.0003), accompanied by a substantial reduction in heterogeneity (*I*^2^ decreased from 89% to 42%). These analyses indicated that this study contributed substantially to the observed heterogeneity.

Publication bias was explored using contour-enhanced funnel plots, Egger's test where appropriate, and trim-and-fill analyses (Supplementary Figures 6–10). For CPSP incidence at 3 months, visual inspection suggested funnel-plot asymmetry. However, Egger's test was not statistically significant (*P* = 0.306), and the trim-and-fill analysis imputed two studies. Trim-and-fill analysis imputed three missing studies for the incidence of neuropathic pain at 3 months, two studies for SF-MPQ scores at 3 months, and one study for CPSP incidence at 6 months. No studies were imputed for pain scores at 3 months, for which Egger's test was also not statistically significant (*P* = 0.148). Given the limited number of studies for several outcomes and the potential for clinical heterogeneity to contribute to funnel-plot asymmetry, these exploratory findings were insufficient to judge publication bias as strongly suspected. The certainty of evidence was assessed using the GRADE framework (Supplementary Table 3). Evidence for the incidence of CPSP at 3 months was rated as moderate, downgraded by one level for risk of bias related to long-term missing outcome data. Evidence for the incidence of CPSP at 6 months was rated as low because of risk of bias and serious imprecision. For the secondary outcomes, evidence for neuropathic pain incidence at 3 months was rated as moderate, downgraded for imprecision because it was based on five small trials with 92 events. Evidence for pain scores at 3 months was rated as low owing to very serious inconsistency, whereas evidence for SF-MPQ scores at 3 months was rated as very low owing to serious risk of bias and very serious inconsistency. Although publication bias could not be excluded, the available assessments were insufficient to judge it as strongly suspected; it was therefore not used as a reason for GRADE downgrading.

### 3.5 Adverse events and safety monitoring

Quantitative analysis was not performed for adverse events because of the scarcity of reported data and inconsistent safety reporting standards across the included studies. Although the overall reported incidence of adverse events associated with perioperative intravenous lidocaine infusion was low, this finding must be interpreted with caution. A structured review of the safety monitoring protocols revealed that many studies relied on passive spontaneous reporting rather than active surveillance for local anesthetic systemic toxicity (LAST).

Trials that employed explicit, active assessment protocols for LAST symptoms (e.g., systematically querying patients for perioral numbness, metallic taste, or lightheadedness) tended to capture a higher incidence of mild, transient neurotoxicity. For instance, Toner et al. reported notably higher rates of lightheadedness (26), metallic taste, and perioral numbness in the lidocaine group. Conversely, studies lacking structured neurologic surveillance frequently reported zero adverse events, suggesting that the true incidence of mild LAST symptoms may be systematically underreported in the literature.

With respect to cardiovascular events, bradycardia and hypotension were documented. Most studies reported no significant difference in hemodynamic stability between the lidocaine and control groups. However, the study by Abdel Rady et al. (45) was a notable exception. They utilized a unique high-dose single infusion of lidocaine (4 mg/kg over 30 min) without a subsequent continuous infusion and reported a markedly higher incidence of hypotension (46.7% vs. 3.3%) and bradycardia (26.7% vs. 3.3%) in the lidocaine group.

Notably, one study [Ibrahim et al. (38)] reported a serious adverse event of generalized convulsion. This occurred during the administration of a 2 mg/kg intravenous bolus. The patient was subsequently withdrawn and excluded from the final efficacy analysis. This incident highlights the risk of systemic toxicity from higher bolus doses or rapid injection rates. Fortunately, most other reported adverse events were transient, self-limiting, and resolved without sequelae. Detailed data on specific adverse events and active surveillance protocols are provided in Supplementary Table 4.

## 4 Discussion

This study integrates current evidence to evaluate the preventive effect of perioperative intravenous lidocaine on CPSP. Our core synthesis suggests a modest but significant reduction in CPSP incidence at 3 months postoperatively. However, this long-term benefit remains uncertain at the 6-month follow-up. While pain intensity outcomes are limited by substantial heterogeneity, the reduction in neuropathic pain incidence demonstrates a promising, albeit limited, signal based on a small number of studies.

The apparent difference between the statistically significant result for CPSP incidence at 3 months and the inconclusive result at 6 months should be interpreted cautiously. Although perioperative intravenous lidocaine was associated with a lower incidence of CPSP at 3 months, the pooled estimate at 6 months was imprecise and did not reach statistical significance. This pattern does not by itself establish a time-dependent loss of treatment effect. Clinical diversity across trials and the substantial heterogeneity observed for pain scores at 3 months may have contributed to the variability in effect estimates.

We conducted exploratory subgroup analyses to examine whether study-level clinical and administration characteristics might explain this variability. For CPSP incidence at 6 months, the pooled estimate within the breast-surgery subgroup favored lidocaine (RR = 0.56, 95% CI: 0.34–0.92). However, this finding was based on four studies, and the test for subgroup differences between breast and non-breast surgery was not statistically significant (*P* = 0.08). It should therefore be interpreted as an outcome-specific, hypothesis-generating observation rather than evidence that patients undergoing breast surgery derive greater benefit than those undergoing other procedures. Across the primary CPSP outcomes, the subgroup analyses did not establish a superior administration period or continuous infusion rate. These exploratory findings should not be used to define an optimal lidocaine regimen and require confirmation in adequately powered randomized trials.

Potential mechanisms may help explain the early association between perioperative intravenous lidocaine and a lower incidence of CPSP at 3 months. The observed benefit is compatible with proposed mechanisms involving sodium-channel blockade and modulation of sensitization, although these mechanisms were not directly evaluated in this meta-analysis (16). Additionally, systemic anti-inflammatory effects likely mitigate the surgery-induced neuroinflammatory cascade (18). The inconclusive estimate at 6 months may reflect limited precision, clinical heterogeneity, and the multifactorial mechanisms contributing to persistent pain, rather than demonstrating a definite loss of treatment effect over time. Such persistent pain may involve established neural remodeling and psychosocial factors (5, 47, 48) that may not be fully reversible by a short-term perioperative intervention alone.

Our meta-analysis revealed that perioperative lidocaine infusion did not significantly reduce the overall incidence of CPSP at 6 months. This finding aligns with recent reviews (21, 49), suggesting limited long-term benefits when applied broadly to unselected surgical populations. However, sensitivity analysis indicated that the pooled results were sensitive to methodological variations. For instance, the study by Beaussier et al. employed a fixed-dose postoperative infusion of 60 mg/h (20). This fixed rate differs from standard weight-based protocols and translates to a notably low effective dose for an average adult. Excluding Beaussier et al. shifted the pooled estimate to statistical significance, indicating sensitivity to this individual study. However, this analysis cannot determine whether its dosing regimen, surgical population, or other study characteristics explain the difference. The overall evidence for CPSP prevention at 6 months therefore remains inconclusive.

### 4.1 Contextualizing efficacy: the impact of background analgesia and pharmacokinetics

A critical yet frequently overlooked factor influencing the apparent efficacy of systemic lidocaine is the baseline analgesic protocol. As summarized in Supplementary Table 2, most trials used systemic opioid or non-opioid multimodal analgesia, and some permitted local infiltration. Background analgesic regimens varied across trials, limiting their formal assessment as effect modifiers. In addition, many of these regimens differ substantially from modern Enhanced Recovery After Surgery (ERAS) pathways, in which regional anesthesia is often a cornerstone. In clinical settings where dense afferent blockade has already been established by regional anesthesia, the systemic anti-inflammatory and sodium-channel-blocking properties of lidocaine might offer only diminishing incremental benefit. Whether lidocaine provides any synergistic or additive prophylactic benefit when patients already receive optimal regional anesthesia remains a critical gap in the current literature. Therefore, evaluating the additive effect of intravenous lidocaine in the context of contemporary regional anesthesia should be a priority for future high-quality trials. Furthermore, the profound methodological heterogeneity observed in our secondary outcomes may partly reflect heterogeneity in lidocaine administration protocols (Supplementary Table 1). The included trials used different initial doses, continuous infusion rates, administration periods, and weight-scaling approaches. Hojski et al. reported dosing based on actual body weight, whereas Toner et al. used total body weight capped at 100 kg. Most remaining trials did not clearly specify the weight-scaling method.

Consequently, the actual systemic exposure achieved by a given nominal regimen remains uncertain and may have varied considerably across studies.

Interindividual pharmacokinetic variability may further contribute to heterogeneous responses to perioperative intravenous lidocaine. Lidocaine is substantially bound to alpha-1-acid glycoprotein (AAG), an acute-phase protein whose concentration may change after surgical trauma. Consequently, total plasma lidocaine concentrations may not consistently reflect unbound lidocaine exposure across surgical patients (50). A uniform weight-based infusion rate, including commonly used regimens of 2 mg/kg/h, may therefore not produce comparable exposure or clinical effects in all individuals. Future pharmacokinetic-pharmacodynamic trials should measure total and unbound lidocaine concentrations together with AAG concentrations and evaluate their relationships with analgesic efficacy and toxicity. Such studies are needed to define clinically meaningful exposure targets and inform individualized dosing strategies.

### 4.2 Pain intensity and clinical significance

The overall analysis of postoperative pain scores at 3 months revealed substantial heterogeneity (*I*^2^ = 89%). Sensitivity analysis identified the study by Abdel Rady et al. as a major outlier (45), which utilized a distinct high-dose (4 mg/kg), 30 min single-infusion regimen. Excluding this outlier reduced the heterogeneity to a moderate level (*I*^2^ = 42%). The pooled reduction in pain scores at 3 months was statistically significant (MD = −0.57 on a 0–10 scale). However, its clinical importance cannot be determined by directly comparing this between-group mean difference with thresholds for clinically important within-patient change (51–53). Moreover, clinically important differences may vary according to the pain instrument, surgical population, baseline pain severity, and the context of between-group comparison. Therefore, this secondary finding should be interpreted cautiously and should not alone be taken as evidence of a clinically important analgesic benefit.

Substantial heterogeneity was observed for pain scores at 3 months. This heterogeneity may reflect differences across trials in surgical populations, pain instruments, assessment conditions (resting vs. movement-evoked pain), follow-up procedures, and co-analgesic regimens; these potential contributors could not be disentangled in the present analysis. In contrast, the pooled estimate for neuropathic pain incidence was statistically significant and showed lower statistical heterogeneity, indicating greater consistency of the observed study-level estimates.

### 4.3 Safety profile and limitations of adverse-event reporting

Although our systematic review identified that serious neurotoxic events were rare, hemodynamic events and mild neurologic symptoms were reported in some trials. Differences in safety surveillance and adverse-event reporting limited comparisons across trials (Supplementary Table 4). Mild symptoms compatible with local anesthetic systemic toxicity may have been underdetected in studies without explicit symptom surveillance. Trials that employed active, explicit surveillance for these symptoms [e.g., Toner et al. (26)] consistently reported a higher incidence of transient neurotoxicity compared to trials relying on standard, passive observation, which frequently reported zero events. However, the extent of underreporting could not be quantified, and differences in reported event rates cannot be attributed to surveillance methods alone.

To avoid systemic toxicity, existing therapeutic frameworks suggest maintaining plasma lidocaine concentrations below 5 µg/mL (54). However, most trials included in this review did not actively measure plasma concentrations. The reported convulsion in one trial (38) and the hemodynamic instability reported by Abdel Rady et al. (45), in which 4 mg/kg lidocaine was administered as a single 30 min infusion without a subsequent continuous infusion, underscore the importance of careful administration and safety surveillance. Future trials should incorporate active surveillance for early manifestations of local anesthetic systemic toxicity, transparent reporting of administration kinetics, and structured pharmacokinetic monitoring to clarify exposure-related safety thresholds.

### 4.4 Comparison with previous meta-analyses

Previous reviews used different statistical approaches. For example, Hussain et al. (22) applied conservative Hartung–Knapp–Sidik–Jonkman (HKSJ) (55) adjustment, which may yield wider confidence intervals in random-effects meta-analysis. In the present review, which incorporated more recent evidence, the protective association for CPSP incidence at 3 months remained statistically significant in the REML-HKSJ sensitivity analysis, whereas the 6-month result remained inconclusive.

Importantly, although their results did not reach statistical significance, their pooled point estimates (OR = 0.53 at 3 months) were entirely consistent with the clinically beneficial trends observed in our study. These findings support a cautious interpretation of the early preventive signal and do not establish sustained long-term benefit.

### 4.5 Limitations

Our findings should be interpreted within the context of several limitations. First, publication bias cannot be completely excluded. The limited number of studies for several outcomes reduced the informativeness of funnel-plot and trim-and-fill analyses, and clinical heterogeneity may have contributed to apparent funnel-plot asymmetry. Second, significant residual heterogeneity persisted in secondary outcomes. Third, the certainty of evidence ranged from moderate to very low, reflecting outcome-specific concerns, including long-term missing outcome data, imprecision, and substantial heterogeneity. Fourth, the decision to exclude acute postoperative pain was made after data extraction had commenced, which may introduce outcome-reporting bias. Although this change did not affect the eligibility criteria or the CPSP analyses, it was a *post hoc* protocol amendment. Finally, most primary studies did not distinguish between the neuropathic and nociceptive components of CPSP.

## 5 Conclusion

Perioperative intravenous lidocaine may offer a modest reduction in the incidence of CPSP and neuropathic pain at 3 months postoperatively. However, evidence for long-term benefits at 6 months remains uncertain, and the certainty of evidence across outcomes ranges from moderate to very low. Exploratory subgroup findings did not establish a superior surgical population, administration period, or continuous infusion rate and should not be used to define an optimal lidocaine regimen. Future trials should use standardized and transparently reported administration protocols, active safety surveillance, structured pharmacokinetic monitoring, and exposure-response analyses to clarify which patients are most likely to benefit.

## Data Availability

The original contributions presented in the study are included in the article/Supplementary Material, further inquiries can be directed to the corresponding author/s.

## Appendix 1: Search strategiesPubMed search strategy

#1. Lidocaine [Mesh]

#2. lidocain* OR Lignocain* OR Xylocain*

#3. #1 OR #2

#4. “infusions, intravenous” [Mesh]

#5. injection OR infusion OR "IV"

#6. #4 OR #5

#7. #3 AND #6

#8. pain [Title/Abstract]

#9. Chronic OR persistent OR Month*

#10. #8 AND #9

#11. Surgery [Mesh]

#12. Operat* OR intraoperative* OR perioperative* OR postop* OR surg*

#13. #11 OR #12

#14. #7 AND #10 AND #13

EMBASE search strategy

#1. “lidocaine”/exp

#2. lidocain*:ab,ti OR lignocain*:ab,ti OR xylocain*:ab,ti

#3. #1 OR #2

#4. “intravenous drug administration”/exp

#5. inject*:ab,ti OR infus*:ab,ti OR iv:ab,ti

#6. #4 OR #5

#7. #3 AND #6

#8. pain:ab,ti

#9. chronic:ab,ti OR persistent:ab,ti OR month*:ab,ti

#10. #8 AND #9

#11. “surgery”/exp

#12. operat*:ab,ti OR intraoperative*:ab,ti OR perioperative*:ab,ti OR postop*:ab,ti OR surg*:ab,ti

#13. #11 OR #12

#14. #7 AND #10 AND #13

Cochrane Library search strategy

#1. MeSH descriptor: [Lidocaine] explode all trees

#2. (lidocain*):ti,ab,kw OR (lignocain*):ti,ab,kw OR (xylocain*):ti,ab,kw

#3. #1 OR #2

#4. MeSH descriptor: [Injections, Intravenous] explode all trees

#5. (injec*):ti,ab,kw OR (infus*):ti,ab,kw OR (IV):ti,ab,kw

#6. #4 OR #5

#7. #3 AND #6

#8. (pain):ti,ab,kw

#9. (Chronic):ti,ab,kw OR (persistent):ti,ab,kw OR (Month*):ti,ab,kw

#10. #8 AND #9

#11. MeSH descriptor: [General Surgery] explode all trees

#12. (Operat*):ti,ab,kw OR (intraoperative*):ti,ab,kw OR (perioperative*):ti,ab,kw OR (postop*):ti,ab,kw OR (surg*):ti,ab,kw

#13. #11 OR #12

#14. #7 AND #10 AND #13
