# Supplementary material for: Perioperative intravenous lidocaine for the prevention of chronic postsurgical pain: a systematic review and meta-analysis of randomized controlled trials
Source: Front Med (Lausanne). 2026 Sep 28;13:1927770. doi: 10.3389/fmed.2026.1927770 (PMC13654987; doi:10.3389/fmed.2026.1927770)
Supplement: Supplementary file 1 [file Datasheet1.pdf]

Appendix 1: CENTRAL search strategy (PubMed)

#1. Lidocaine [Mesh]

#2. lidocain* OR Lignocain* OR Xylocain*

#3. #1 OR #2

#4. “infusions, intravenous" [Mesh]

#5. injection OR infusion OR "IV"

#6. #4 OR #5

#7. #3 AND #6

#8. pain [Title/Abstract]

#9. Chronic OR persistent OR Month*

#10. #8 AND #9

#11. Surgery [Mesh]

#12. Operat* OR intraoperative* OR perioperative* OR postop* OR surg*

#13. #11 OR #12

#14. #7 AND #10 AND #13

CENTRAL search strategy (EMBASE)

#1. ‘lidocaine’/exp

#2. lidocain*:ab,ti OR lignocain*:ab,ti OR xylocain*:ab,ti

#3. #1 OR #2

#4. ‘intravenous drug administration’/exp

#5. inject*:ab,ti OR infus*:ab,ti OR iv:ab,ti

#6. #4 OR #5

#7. #3 AND #6

#8. pain:ab,ti

#9. chronic:ab,ti OR persistent:ab,ti OR month*:ab,ti

#10. #8 AND #9

#11. ‘surgery’/exp

#12. operat*:ab,ti OR intraoperative*:ab,ti OR perioperative*:ab,ti OR postop*:ab,ti OR

surg*:ab,ti

#13. #11 OR #12

#14. #7 AND #10 AND #13

CENTRAL search strategy (Cochrane Library)

#1. MeSH descriptor: [Lidocaine] explode all trees

#2. (lidocain*):ti,ab,kw OR (lignocain*):ti,ab,kw OR (xylocain*):ti,ab,kw

#3. #1 OR #2

#4. MeSH descriptor: [Injections, Intravenous] explode all trees

#5. (injec*):ti,ab,kw OR (infus*):ti,ab,kw OR (IV):ti,ab,kw

#6. #4 OR #5

#7. #3 AND #6

#8. (pain):ti,ab,kw

#9. (Chronic):ti,ab,kw OR (persistent):ti,ab,kw OR (Month*):ti,ab,kw

#10. #8 AND #9

#11. MeSH descriptor: [General Surgery] explode all trees

#12. (Operat*):ti,ab,kw OR (intraoperative*):ti,ab,kw OR (perioperative*):ti,ab,kw OR (postop*):ti,ab,kw OR (surg*):ti,ab,kw

#13. #11 OR #12

#14. #7 AND #10 AND #13
