# Supplementary material for: Perioperative intravenous lidocaine for the prevention of chronic postsurgical pain: a systematic review and meta-analysis of randomized controlled trials
Source: Front Med (Lausanne). 2026 Sep 28;13:1927770. doi: 10.3389/fmed.2026.1927770 (PMC13654987; doi:10.3389/fmed.2026.1927770)
Supplement: Supplementary file 2 [file Datasheet2.pdf]

Supplementary Materials

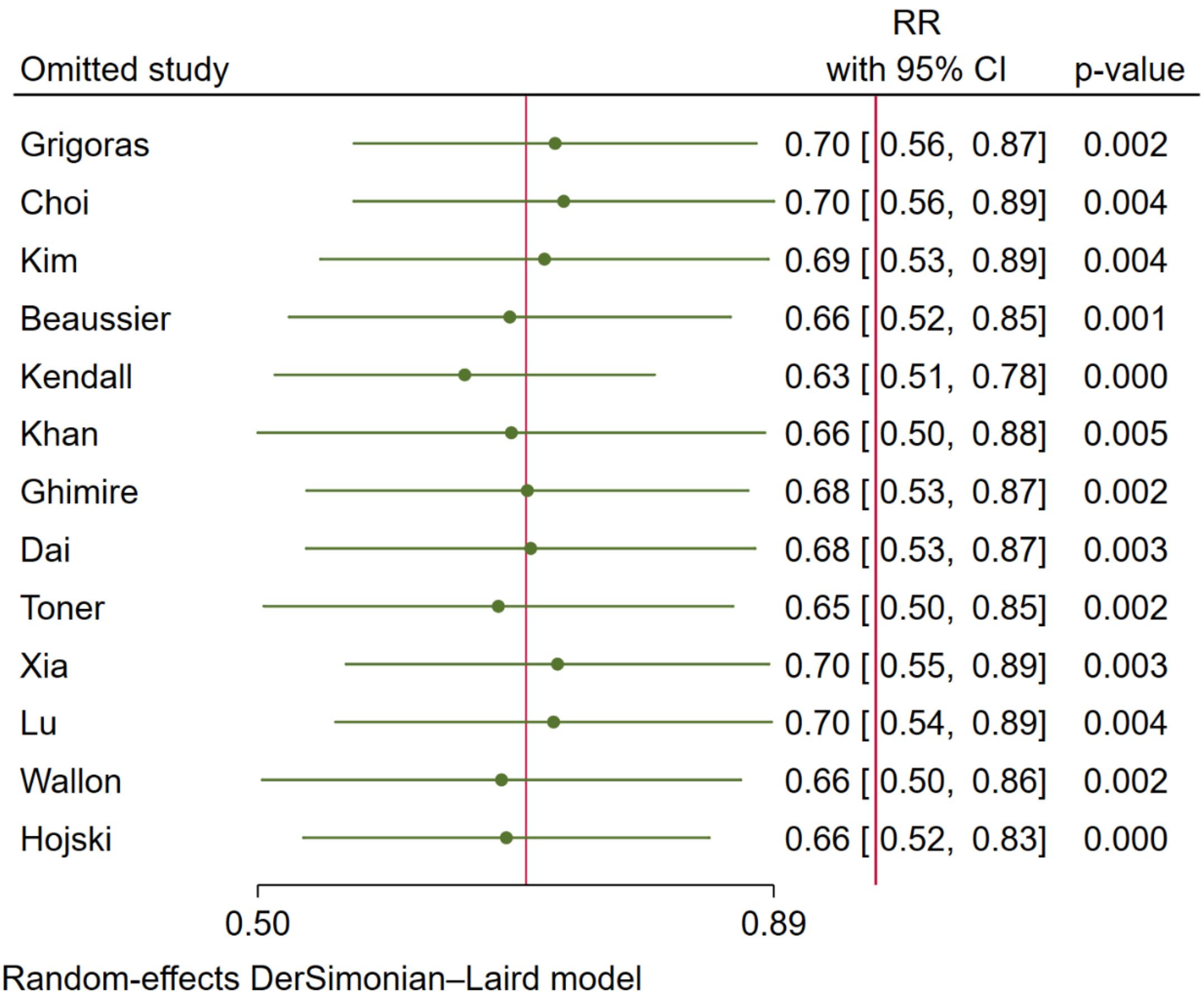

Supplementary figure 1 - Sensitivity Analysis of the Incidence of CPSP at 3 Months.

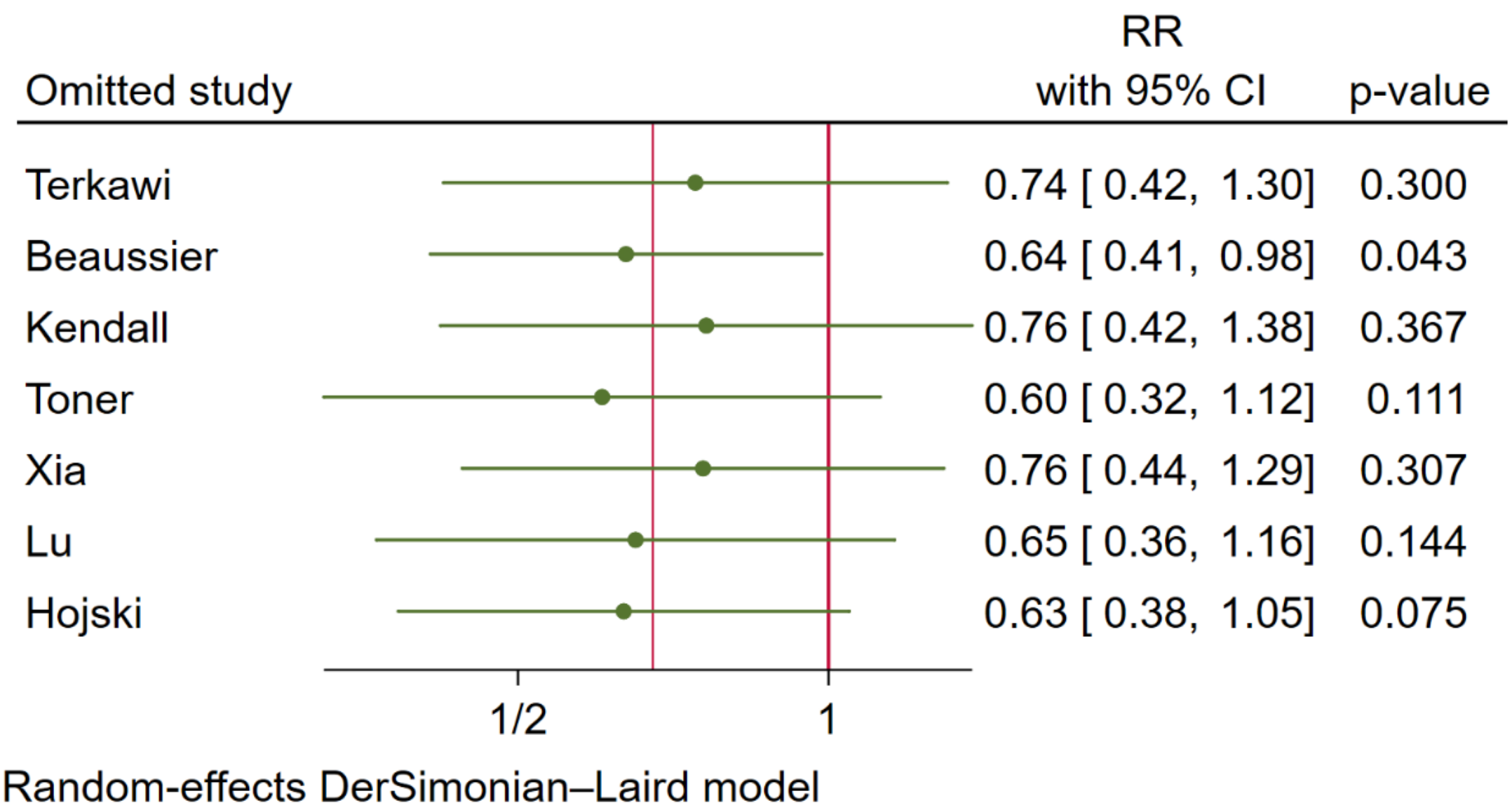

Supplementary figure 2 - Sensitivity Analysis of the Incidence of CPSP at 6 Months.

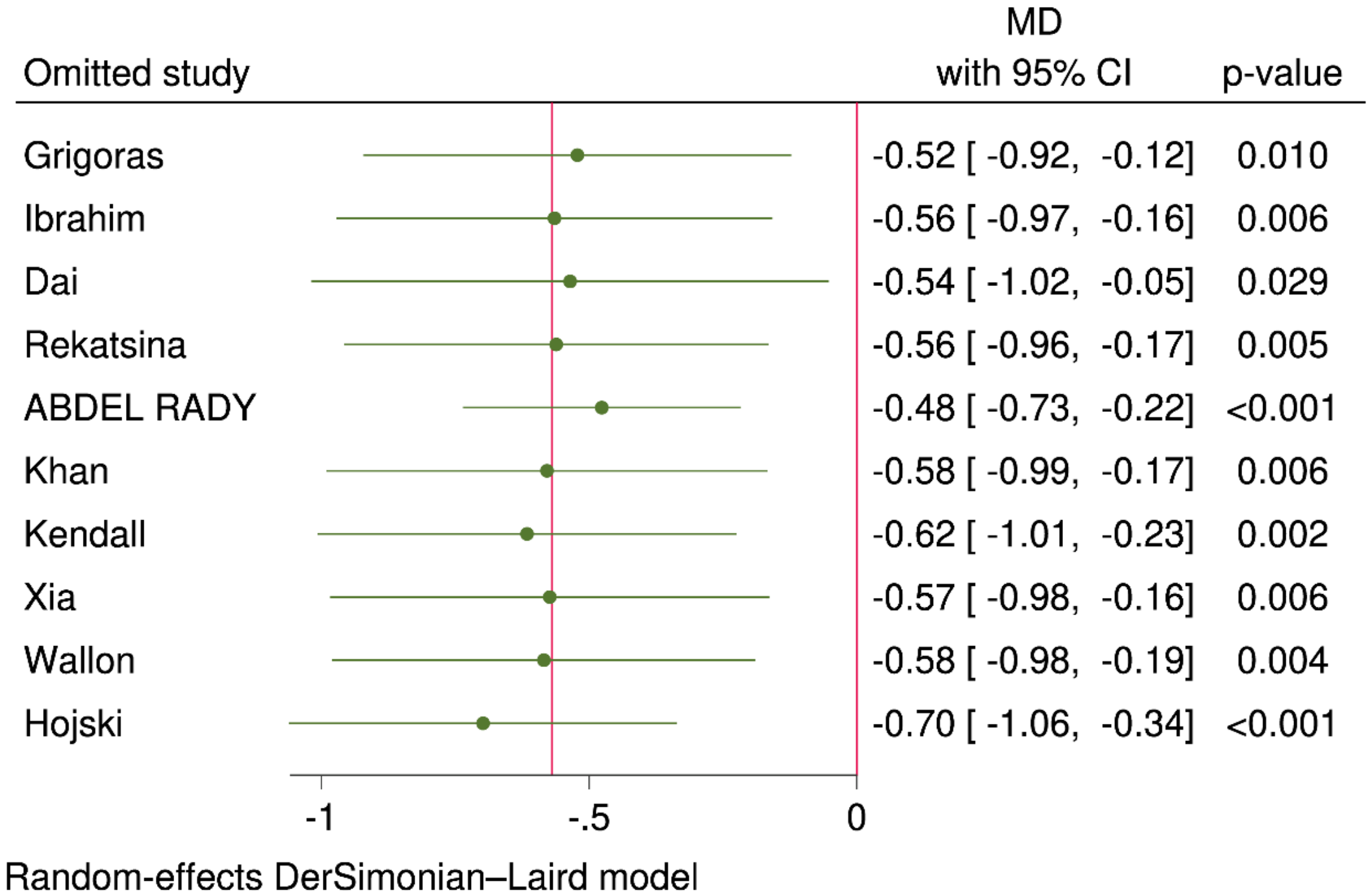

Supplementary figure 3 - Sensitivity Analysis of the pain scores at 3 Months.

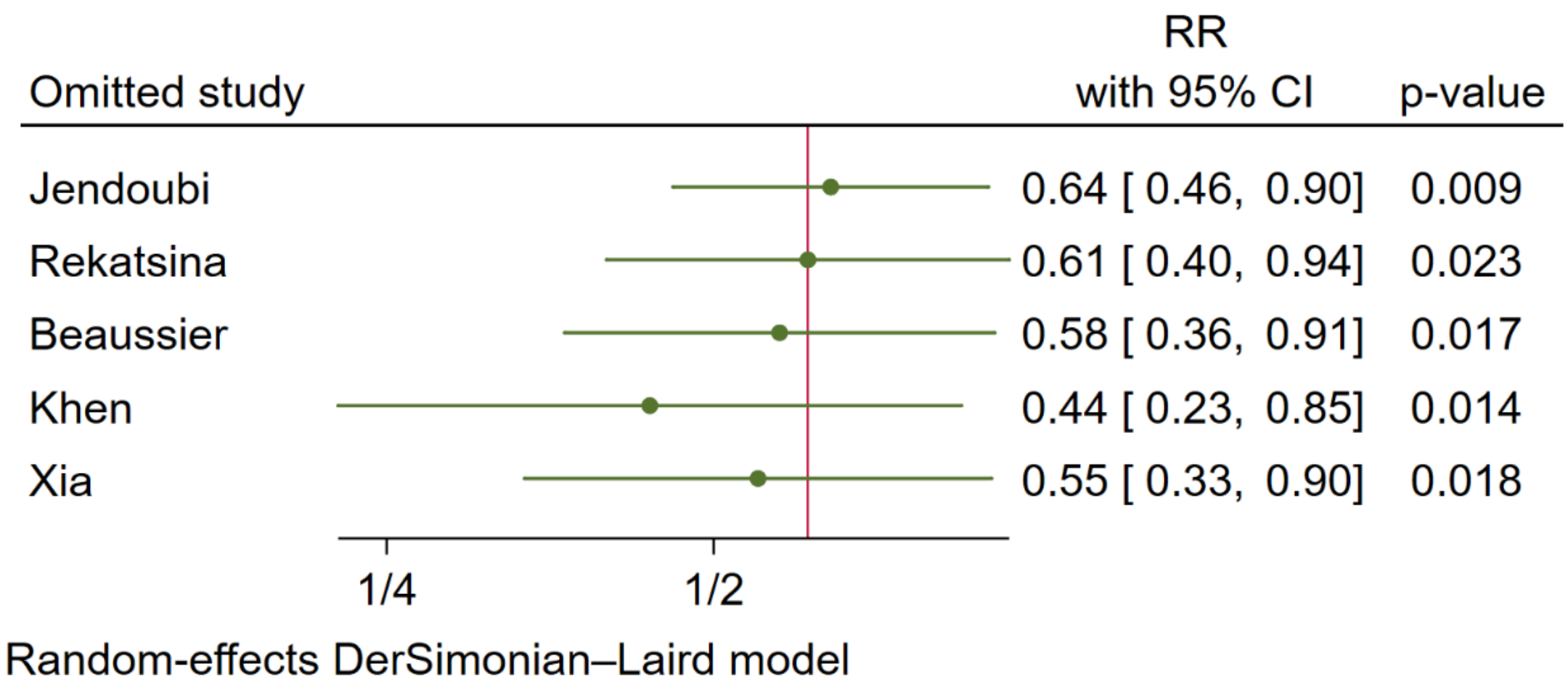

Supplementary figure 4 - Sensitivity Analysis of the Incidence of Neuropathic at 3 Months.

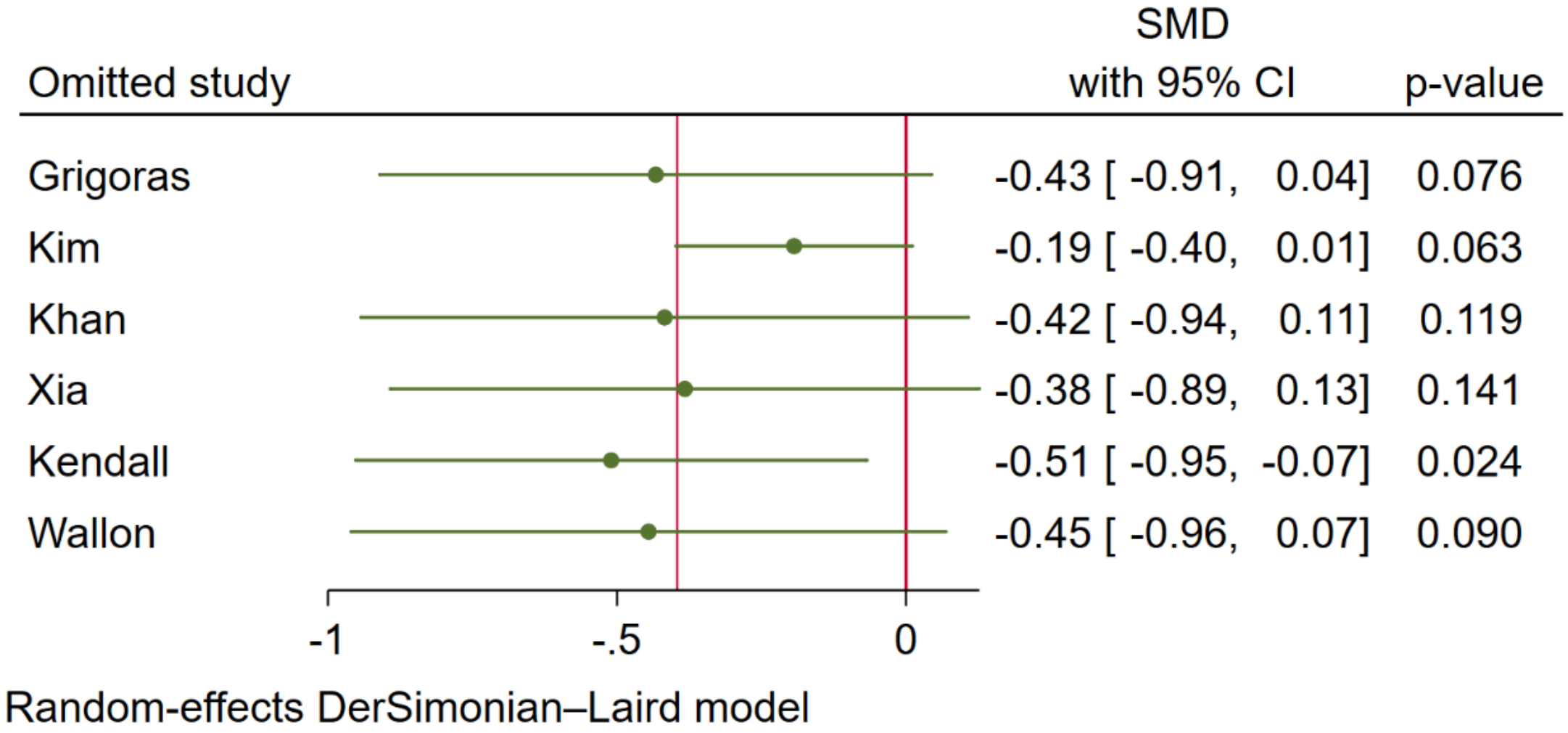

Supplementary figure 5 - Sensitivity Analysis of the SF-MPQ scores at 3 Months.
