# Supplementary material for: Perioperative intravenous lidocaine for the prevention of chronic postsurgical pain: a systematic review and meta-analysis of randomized controlled trials
Source: Front Med (Lausanne). 2026 Sep 28;13:1927770. doi: 10.3389/fmed.2026.1927770 (PMC13654987; doi:10.3389/fmed.2026.1927770)
Supplement: Supplementary file 3 [file Datasheet3.pdf]

Supplementary Materials

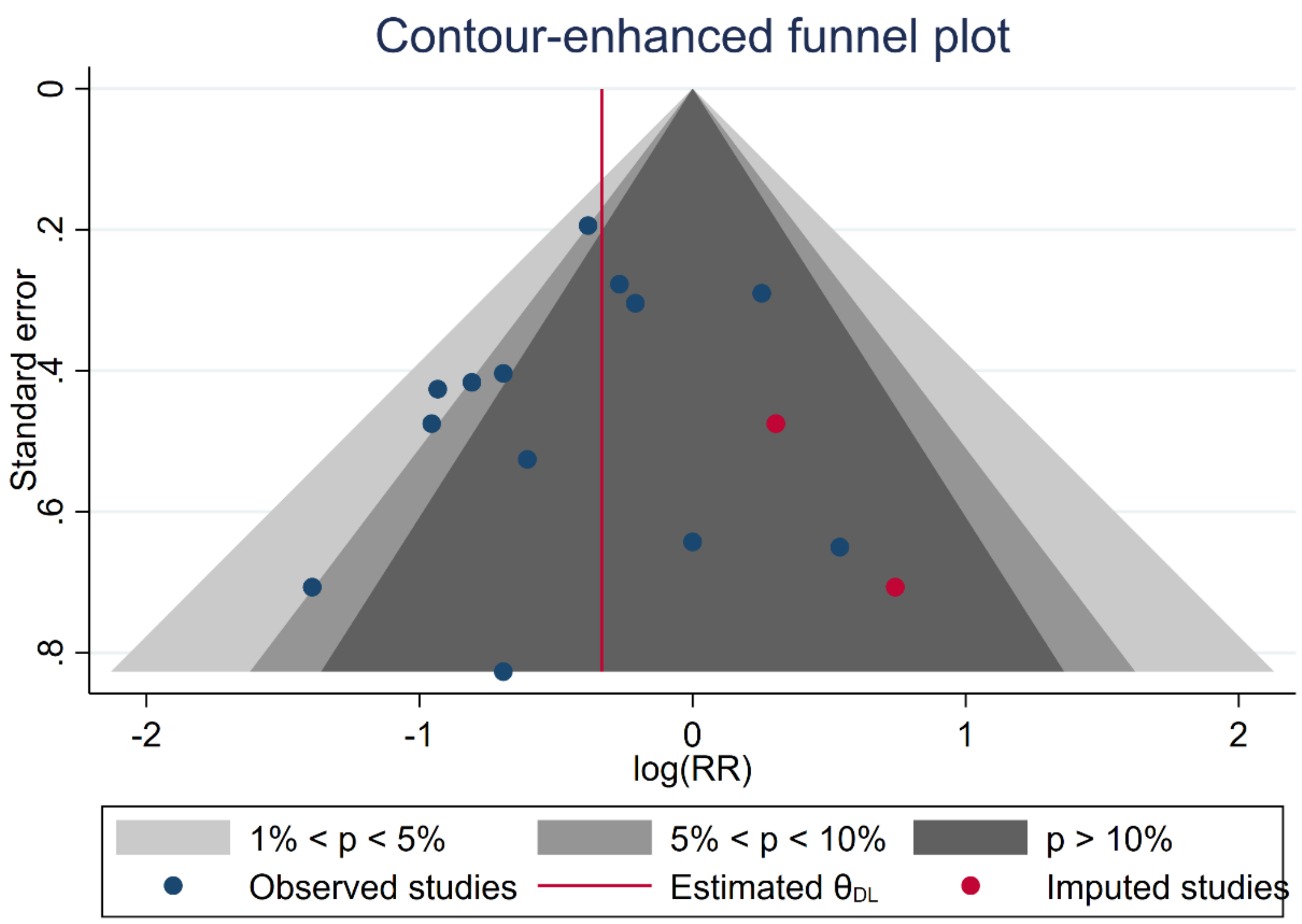

Supplementary figure 6 - Trim and fill method for enhanced funnel plot of the incidence of CPSP at 3 months.

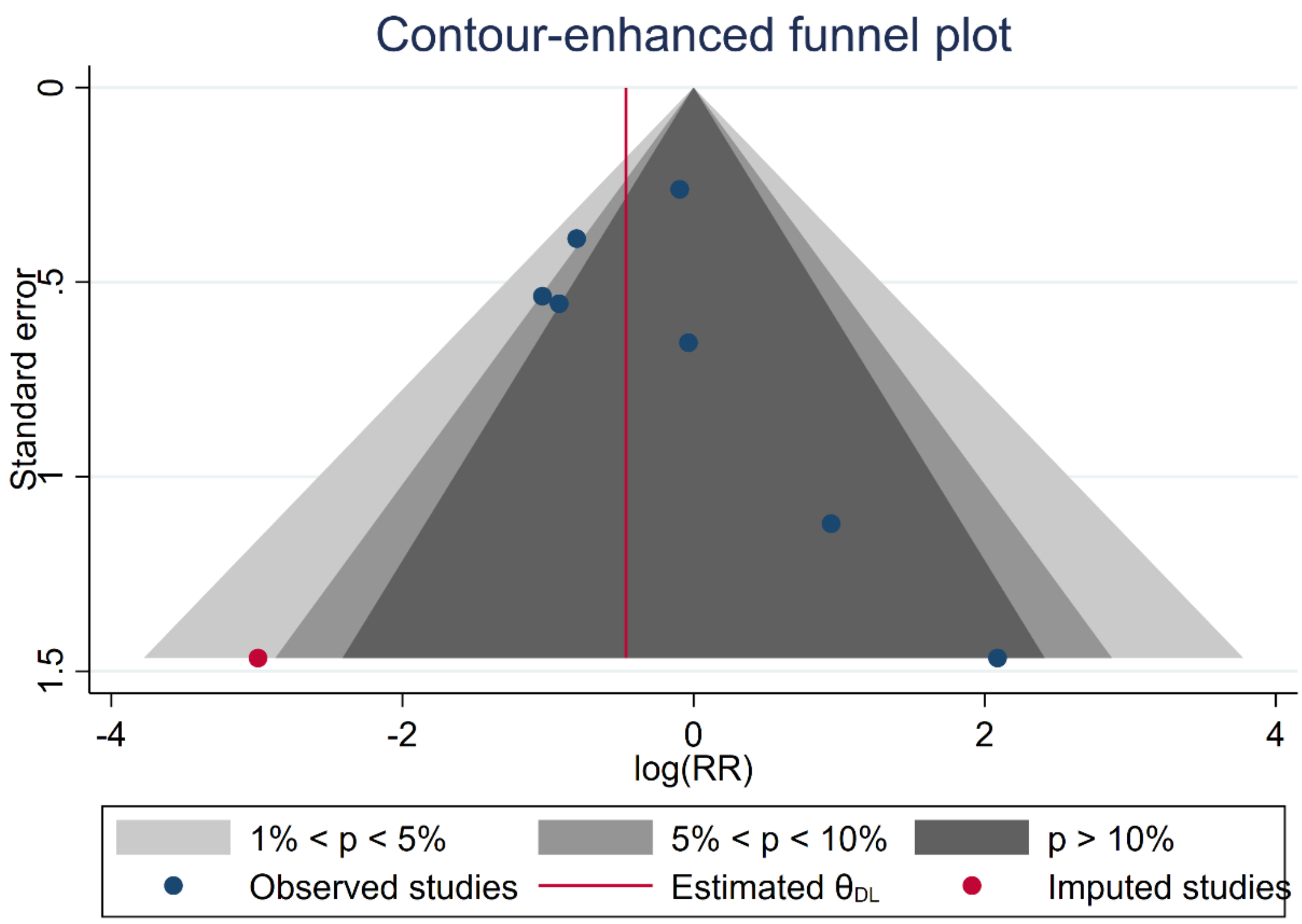

Supplementary figure 7 - Trim and fill method for enhanced funnel plot of the incidence of CPSP at 6 months.

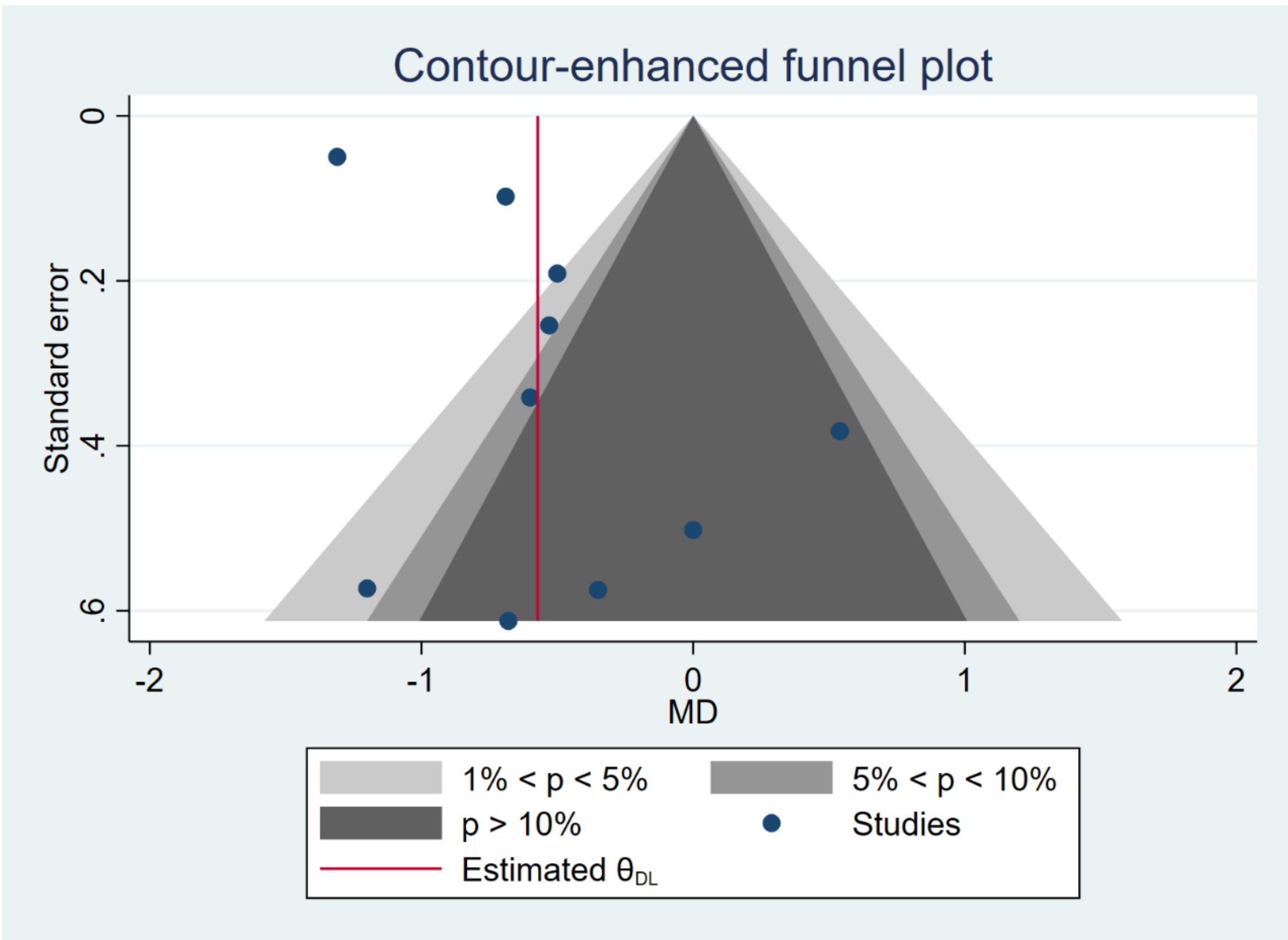

Supplementary figure 8 - Trim and fill method for enhanced funnel plot of the pain scores at 3 months.

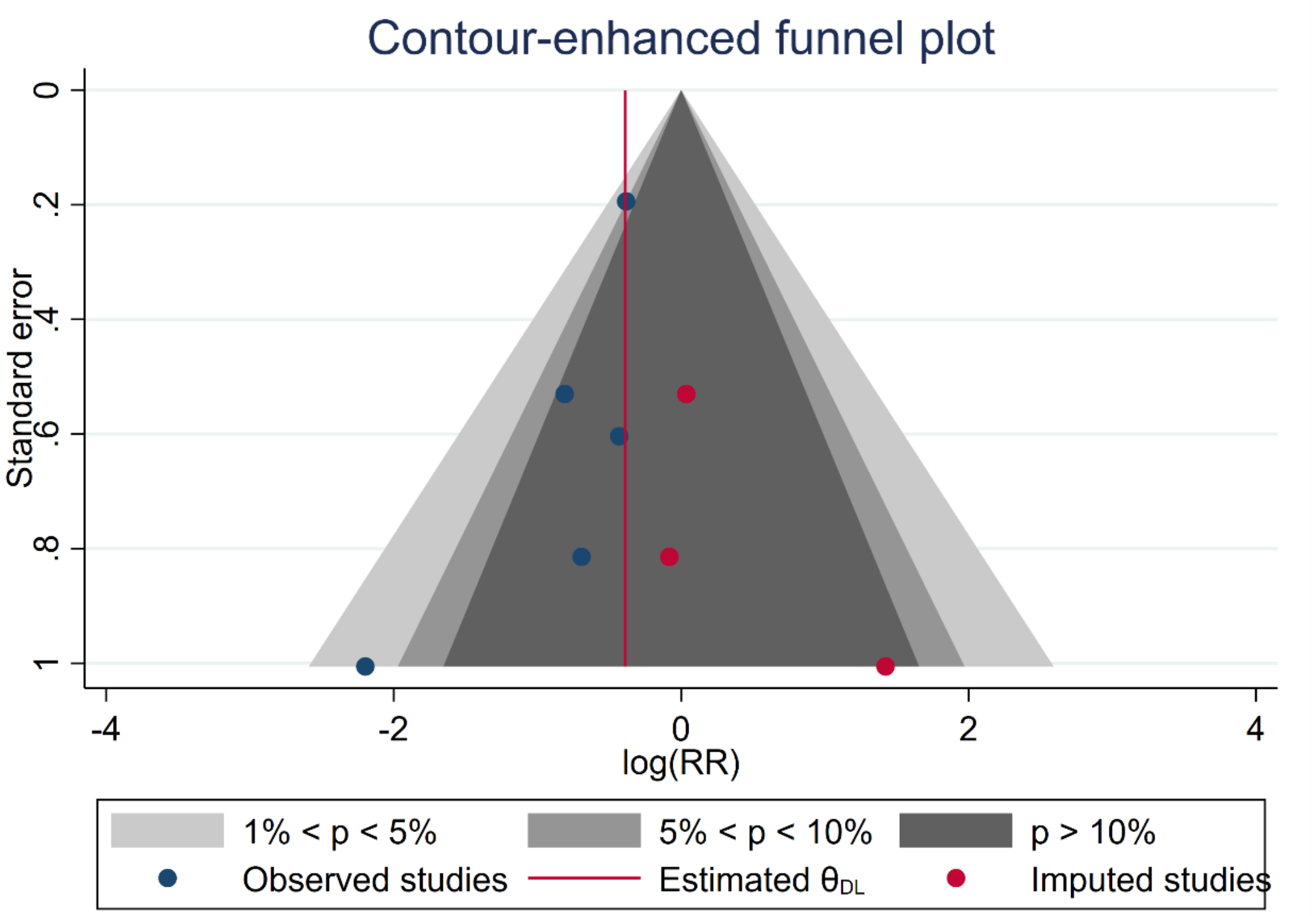

Supplementary figure 9 - Trim and fill method for enhanced funnel plot of the incidence of Neuropathic at 3 months.

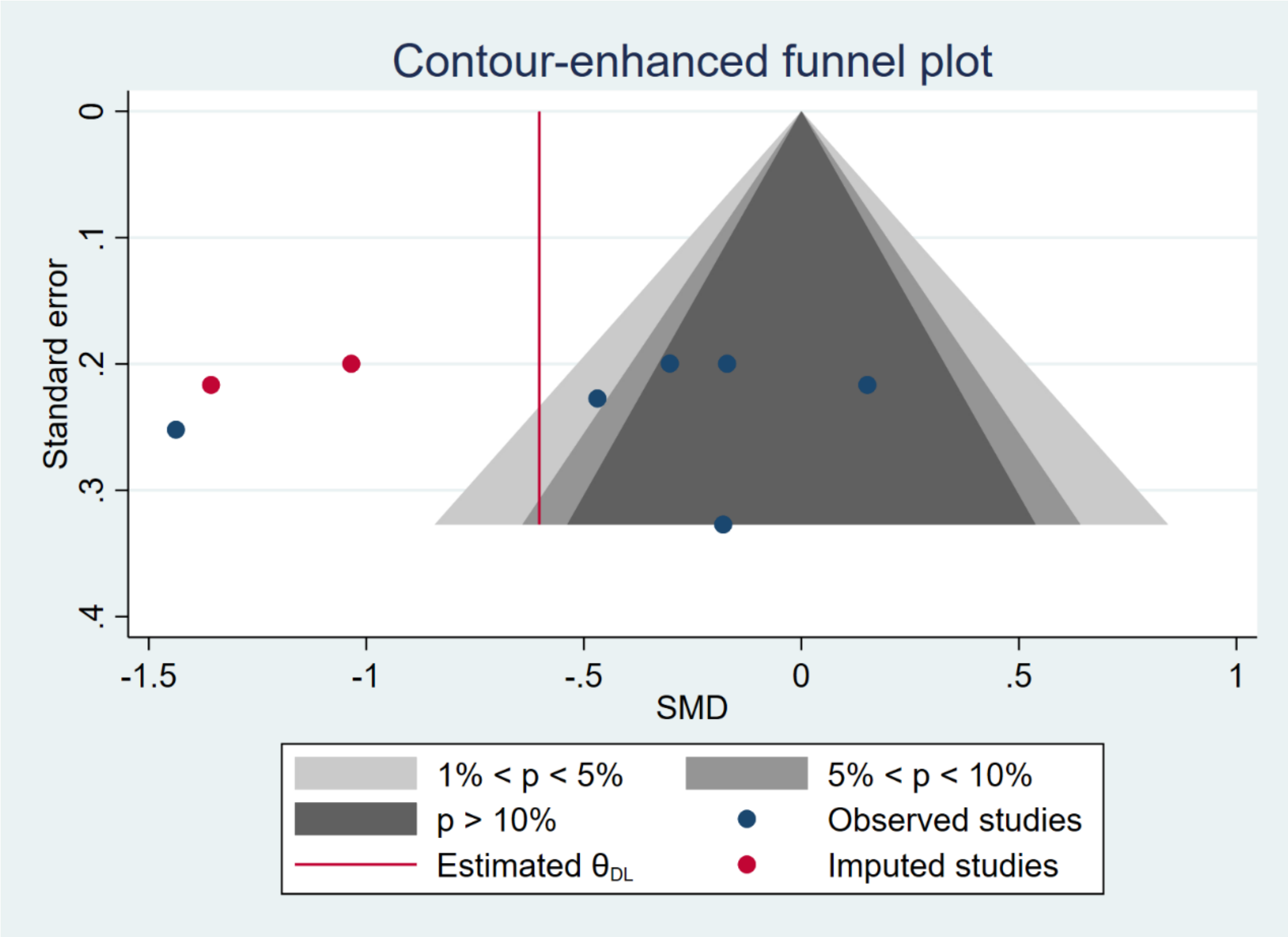

Supplementary figure 10 - Trim and fill method for enhanced funnel plot of the SF-MPQ scores at 3 months.
