# Supplementary material for: Perioperative intravenous lidocaine for the prevention of chronic postsurgical pain: a systematic review and meta-analysis of randomized controlled trials
Source: Front Med (Lausanne). 2026 Sep 28;13:1927770. doi: 10.3389/fmed.2026.1927770 (PMC13654987; doi:10.3389/fmed.2026.1927770)
Supplement: Supplementary file 4 [file Datasheet4.pdf]

Supplementary Materials

Study	Experimental Events	Experimental Total	Control Events	Control Total	RR	95% CI	Weight
Beaussier	4	23	4	23	1.00	[0.28; 3.52]	3.2%
Choi	6	41	16	43	0.39	[0.17; 0.91]	6.7%
Dai	5	58	9	57	0.55	[0.19; 1.53]	4.6%
Ghimire	2	30	4	30	0.50	[0.10; 2.53]	2.0%
Grigoras	2	17	9	19	0.25	[0.06; 0.99]	2.7%
Hojski	6	28	3	24	1.71	[0.48; 6.13]	3.1%
Kendall	18	43	13	40	1.29	[0.73; 2.27]	12.4%
Khan	22	51	31	49	0.68	[0.47; 1.00]	20.9%
Kim	7	39	14	39	0.50	[0.23; 1.10]	7.3%
Lu	6	29	13	28	0.45	[0.20; 1.01]	6.9%
Toner	15	74	19	76	0.81	[0.45; 1.47]	11.5%
Wallon	14	43	20	47	0.77	[0.44; 1.32]	13.2%
Xia	5	39	13	39	0.38	[0.15; 0.98]	5.5%
**REML−HKSJ random−effects model**		**515**		**514**	**0.68**	**[0.52; 0.88]**	**100.0%**

Heterogeneity: $I^2 = 18.8\%$, $\tau^2 = 0.0298$, $p = 0.2540$

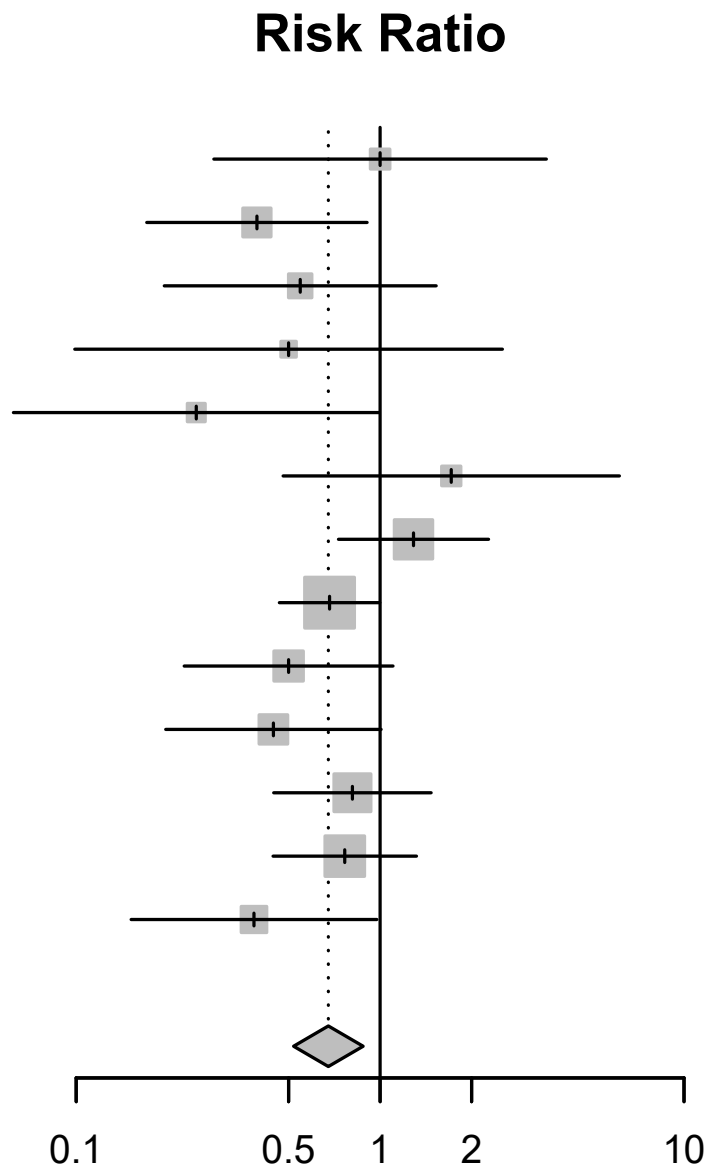

Supplementary figure 11 - Hartung-Knapp-Sidik-Jonkman sensitivity analysis for the incidence of CPSP at 3 months.

Study	Experimental Events	Experimental Total	Control Events	Control Total	RR	95% CI	Weight
Beaussier	4	28	0	25	8.05	[0.46; 142.38]	2.2%
Hojski	3	28	1	24	2.57	[0.29; 23.13]	3.7%
Kendall	8	62	17	59	0.45	[0.21; 0.96]	22.2%
Lu	4	29	4	28	0.97	[0.27; 3.49]	9.9%
Terkawi	4	34	8	27	0.40	[0.13; 1.18]	13.0%
Toner	20	74	22	74	0.91	[0.54; 1.52]	35.1%
Xia	4	38	11	37	0.35	[0.12; 1.01]	13.8%
**REML−HKSJ random−effects model**		**293**		**274**	**0.67**	**[0.36; 1.24]**	**100.0%**

Heterogeneity: $I^2 = 35.8\%$, $\tau^2 = 0.0732$, $p = 0.1553$

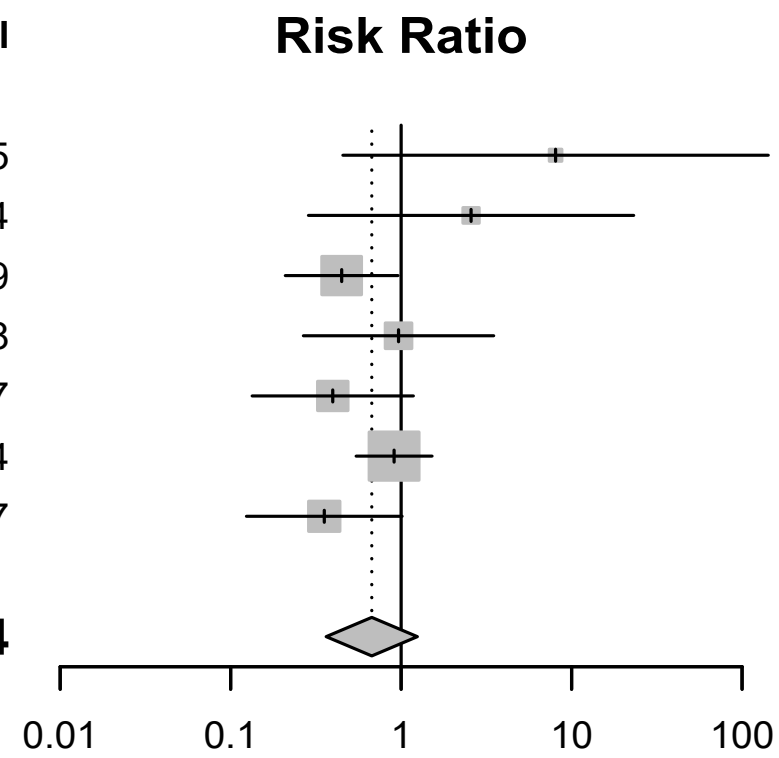

Supplementary figure 12 - Hartung-Knapp-Sidik-Jonkman sensitivity analysis for the incidence of CPSP at 6 months.
