# Supplementary material for: Perioperative intravenous lidocaine for the prevention of chronic postsurgical pain: a systematic review and meta-analysis of randomized controlled trials
Source: Front Med (Lausanne). 2026 Sep 28;13:1927770. doi: 10.3389/fmed.2026.1927770 (PMC13654987; doi:10.3389/fmed.2026.1927770)
Supplement: Supplementary file 6 [file Table1.docx]

**Supplementary table 1**

Detailed Protocols for Lidocaine Administration and Pharmacokinetic Monitoring.

| Study | Bolus/Single-Infusion Administration Duration | Weight-Scaling Method | Dose-Adjustment / Discontinuation Criteria | Plasma Level Monitoring |
| --- | --- | --- | --- | --- |
| Grigoras (2012) | Over 10 min | Not reported | None described | Yes (venous blood at 60 min after skin closure.) |
| Terkawi (2015) | Unspecified bolus | Not reported (max dose 150 mg) | None described | No |
| Choi (2016) | Over 10 min | Not reported | None described | No |
| Kim (2017) | Over 15 min | Not reported | None described | No |
| Jendoubi (2017) | Unspecified bolus | Not reported | None described | No |
| Beaussier (2018) | Unspecified (Postop only) | Fixed dose (60 mg bolus, 60 mg/h) | None described | No |
| Kendall (2018) | Unspecified bolus | Not reported (max total dose 1200 mg) | None described | No |
| Ibrahim (2018) | Slow IV bolus | Not reported | None described | No |
| Khan (2019) | Unspecified bolus | Not reported | None described | No |
| Ghimire (2020) | Unspecified bolus | Not reported | None described | No |
| Dai (2020) | Slow IV bolus | Not reported | None described | No |
| Toner (2021) | Over 5 min | Total body weight (capped at 100 kg) | Ceased and unblinded if signs of severe toxicity develop. | Yes (venous blood, HPLC at multiple timepoints.) |
| Xia (2022) | Unspecified bolus | Not reported | None described | No |
| Lu (2022) | Over 10 s | Not reported | None described | No |
| Wallon (2022) | Unspecified bolus | Not reported | Stopped if serious adverse events (dysgeusia, tinnitus, seizures, bradycardia <45 bpm, AV block, ventricular arrhythmias) occurred. | No |
|  |  |  | (*continued on next page*) | |

**Supplementary table 1 (*continued*)**

| Study | Bolus/Single-Infusion Administration Duration | Weight-Scaling Method | Dose-Adjustment / Discontinuation Criteria | Plasma Level Monitoring |
| --- | --- | --- | --- | --- |
| Rekatsina (2022) | Over 10 min | Not reported | Not described a priori; one discontinuation for ventricular ectopic beats reported. | No |
| Hojski (2024) | Unspecified bolus | Actual body weight | Not described a priori, Stopped for suspected LAST (one case: metallic taste, bradycardia, hypotension.) | No routine monitoring; serum lidocaine was measured after suspected toxicity in one patient. |
| Abdel Rady (2024) | Over 30 min (single infusion; no subsequent continuous infusion.) | Not reported | None described | No |

HPLC = High-performance liquid chromatography; LC-MS/MS = Liquid chromatography-tandem mass spectrometry; FPIA = Fluorescence polarization immunoassay; IBW = Ideal body weight; ABW = Actual body weight; LAST = Local Anesthetic Systemic Toxicity; HR = Heart rate; SBP = Systolic blood pressure.
