# Supplementary material for: Perioperative intravenous lidocaine for the prevention of chronic postsurgical pain: a systematic review and meta-analysis of randomized controlled trials
Source: Front Med (Lausanne). 2026 Sep 28;13:1927770. doi: 10.3389/fmed.2026.1927770 (PMC13654987; doi:10.3389/fmed.2026.1927770)
Supplement: Supplementary file 7 [file Table2.docx]

**Supplementary table 2**

Summary of Perioperative Analgesic Protocols Across Included Trials.

| Study | Surgical Procedure | Intraoperative Analgesia | Postoperative / Background Analgesia | Peripheral Nerve Blocks Used |
| --- | --- | --- | --- | --- |
| Grigoras (2012) | Breast Surgery | Fentanyl + Paracetamol + Diclofenac + Morphine | Paracetamol, Morphine (rescue) | No |
| Terkawi (2015) | Breast Surgery | Fentanyl | Not standardized / Fentanyl or Morphine PRN | No |
| Choi (2016) | Robotic Thyroidectomy | Remifentanil + Propacetamol | Fentanyl/Tramadol PRN + Ibuprofen | No |
| Kim (2017) | Breast Surgery | Remifentanil + Propacetamol + Nefopam | Not standardized | No |
| Jendoubi (2017) | Open Nephrectomy | Fentanyl + Paracetamol + Nefopam | Morphine PCA + Paracetamol + Nefopam | No |
| Beaussier (2018) | Laparoscopic Colorectal | Sufentanil | Morphine PCA + Paracetamol | No |
| Kendall (2018) | Breast Surgery | Remifentanil + Hydromorphone | Hydromorphone PCA + Hydrocodone/Acetaminophen | No |
| Ibrahim (2018) | Spinal Fusion | Fentanyl + Ketorolac + Paracetamol | Ketorolac + Paracetamol, Morphine (rescue) | No |
| Khan (2019) | Breast Surgery | Not standardized | Not standardized | No (Local infiltration permitted) |
| Ghimire (2020) | Laparoscopic Hernia | Fentanyl + Paracetamol | Ketorolac, Morphine/Tramadol PRN | No (Local infiltration permitted) |
| Dai (2020) | GI Tumor Surgery | Sufentanil | Sufentanil PCA, Tramadol PRN | No |
| Toner (2021) | Breast Surgery | Not standardized | Not standardized | No (Local infiltration permitted) |
| Xia (2022) | Breast Surgery | Sufentanil + Remifentanil | Flurbiprofen Axetil, Fentanyl PRN | No |
| Lu (2022) | Thoracic Surgery | Sufentanil + Remifentanil | Sufentanil PCA (Mixed with Lidocaine in L group) + Dezocine (rescue) | No |
| Wallon (2022) | Head & Neck Cancer | Remifentanil | Paracetamol + Morphine PCA | No |
| Rekatsina (2022) | Gynecological Surgery | Fentanyl | Morphine PCA + Paracetamol + Parecoxib (rescue) | No |
| Hojski (2024) | Thoracic Surgery | Fentanyl + Remifentanil + Metamizole | Paracetamol + NSAIDs + Morphine PCA | No |
| Abdel Rady (2024) | Spine Fusion | Fentanyl + Ketorolac | Ketorolac + Paracetamol, Morphine (rescue) | No |

Table Footnote: PRN= pro re nata; PCA = Patient-Controlled Analgesia; NSAIDs = Nonsteroidal Anti-inflammatory Drugs.

Intraoperative analgesia includes medications administered during anesthetic induction.
