# Supplementary material for: Perioperative intravenous lidocaine for the prevention of chronic postsurgical pain: a systematic review and meta-analysis of randomized controlled trials
Source: Front Med (Lausanne). 2026 Sep 28;13:1927770. doi: 10.3389/fmed.2026.1927770 (PMC13654987; doi:10.3389/fmed.2026.1927770)
Supplement: Supplementary file 8 [file Table3.docx]

**Supplementary table 3**

Certainty of Evidence for Outcomes Assessed Using GRADE.

| **Certainty assessment** | | | | | | | **№ of patients** | | **Effect** | | **Certainty** | **Importance** |
| --- | --- | --- | --- | --- | --- | --- | --- | --- | --- | --- | --- | --- |
| **№ of studies** | **Study design** | **Risk of bias** | **Inconsistency** | **Indirectness** | **Imprecision** | **Other considerations** | **lidocaine** | **placebo** | **Relative (95% CI)** | **Absolute (95% CI)** |  |  |
| **The incidence of CPSP at 3 months** | | | | | | | | | | | | |
| 13 | randomised trials | serious^a^ | not serious | not serious | not serious | none | 112/515 (21.7%) | 168/514 (32.7%) | **RR 0.67** (0.53 to 0.85) | **108 fewer per 1,000** (from 154 fewer to 49 fewer) | ⨁⨁⨁◯ Moderate^a^ |  |
| **The incidence of CPSP at 6 months** | | | | | | | | | | | | |
| 7 | randomised trials | serious^b^ | not serious | not serious | serious^c^ | none | 47/293 (16.0%) | 63/274 (23.0%) | **RR 0.68** (0.41 to 1.12) | **74 fewer per 1,000** (from 136 fewer to 28 more) | ⨁⨁◯◯ Low^b,c^ |  |
| **The incidence of Neuropathic pain at 3 months** | | | | | | | | | | | | |
| 5 | randomised trials | not serious | not serious | not serious | serious^d^ | none | 33/158 (20.9%) | 59/155 (38.1%) | **RR 0.60** (0.43 to 0.85) | **152 fewer per 1,000** (from 217 fewer to 57 fewer) | ⨁⨁⨁◯ Moderate^d^ |  |
| **The pain scores at 3 months** | | | | | | | | | | | | |
| 10 | randomised trials | not serious | very serious^e^ | not serious | not serious | none | 354 | 351 | - | MD **0.57 lower** (0.95 lower to 0.19 lower) | ⨁⨁◯◯ Low^e^ |  |
| **SF-MPQ scores at 3 months** | | | | | | | | | | | | |
| 6 | randomised trials | serious^f^ | very serious^g^ | not serious | not serious | none | 239 | 236 | - | SMD **0.4 SD lower** (0.81 lower to 0.02 higher) | ⨁◯◯◯ Very low^f,g^ |  |

**CI:** confidence interval; **MD:** mean difference; **RR:** risk ratio; **SMD:** standardised mean difference

#### Explanations

a. Downgraded one level for risk of bias because several studies had concerns related to long-term missing outcome data, including a study with a 56% response rate at 3 months.

b. Downgraded one level for risk of bias because missing outcome data and complete-case analyses were common across the included studies.

c. Downgraded one level for imprecision because the confidence interval included clinically important benefit, no important effect, and possible harm.

d. Downgraded one level for imprecision because the estimate was based on only 92 events across five small trials, and the 95% confidence interval ranged from a modest to a large relative reduction in risk.

e. Downgraded two levels for very serious inconsistency because of substantial heterogeneity (I² = 89%) and variability in surgical populations and pain-assessment approaches.

f. Downgraded one level for risk of bias because two studies contributing to this outcome had high risk of bias owing to substantial missing outcome data, and several others had some concerns.

g. Downgraded two levels for very serious inconsistency because of substantial heterogeneity (I² = 80%) and clinical heterogeneity across surgical populations.
