# Supplementary material for: Perioperative intravenous lidocaine for the prevention of chronic postsurgical pain: a systematic review and meta-analysis of randomized controlled trials
Source: Front Med (Lausanne). 2026 Sep 28;13:1927770. doi: 10.3389/fmed.2026.1927770 (PMC13654987; doi:10.3389/fmed.2026.1927770)
Supplement: Supplementary file 9 [file Table4.docx]

**Supplementary Table 4**

Structured summary of safety monitoring protocols and adverse events.

| Study | Safety Monitoring Protocol | Active Assessment for LAST Symptoms | Adverse events possibly related to lidocaine (Lidocaine Group) | Corresponding adverse events in the control group | Other Postoperative Adverse Events (Lidocaine Group) | Other Postoperative Adverse Events (Control Group) |
| --- | --- | --- | --- | --- | --- | --- |
| Grigoras, 2012 | Standard monitoring | Yes (Patients explicitly queried for symptoms) | No lidocaine-related toxicity | Not reported | PONV: 1/17 (5.9%) | PONV: 1/19 (5.3%) |
| Terkawi, 2015 | Standard monitoring (Institutional PACU protocol) | Unspecified (Method not detailed) | No adverse or unintended effects were observed. | Not reported | No severe adverse events | Lymphedema: 1/27 (3.7%) |
| Choi, 2016 | Standard monitoring + BIS + EtCO_2_ | Unspecified (Method not detailed) | No adverse or unintended effects were observed. | Not reported | Rescue antiemetic required in PACU: 0/41 | Rescue antiemetic required in PACU: 9/43 (20.9%) |
| Kim, 2017 | Standard monitoring + BIS | Not clearly described (Relied on patient complaints) | No adverse or unintended effects were observed. | Not reported | No significant difference | No significant difference |
| Jendoubi, 2017 | Standard monitoring + EtCO_2_ | Unspecified (Method unclear) | No lidocaine-related adverse effects. | Not reported | PONV: 0/20 | PONV: 15/20 (75%) |
| Beaussier, 2018 | Standard monitoring (for 48 h) | Not clearly described (Relied on patient complaints) | Perioral paresthesia, metallic taste (subjective symptoms): 2/29 (6.9%) | Not reported | Surgical site infection (superficial): 3/29 (10.3%) | 1. Severe complications requiring emergency surgery: 2/27 (postoperative peritonitis and lower limb compartment syndrome, 1 each).  2. Surgical site infection (superficial): 1/27 (3.7%). |
| Kendall, 2018 | Standard monitoring (intraop & PACU) | Unspecified (Method unclear) | 0 | Not reported | Not reported | Not reported |
|  |  |  |  |  |  | (*continued on next page*) |

**Supplementary Table 4 (*continued*)**

| Study | Safety Monitoring Protocol | Active Assessment for LAST Symptoms | Adverse events possibly related to lidocaine (Lidocaine Group） | Adverse events possibly related to placebo (Control group) | Other Postoperative Adverse Events (Lidocaine Group) | Other Postoperative Adverse Events (Control Group) |
| --- | --- | --- | --- | --- | --- | --- |
| Ibrahim, 2018 | Standard monitoring + EtCO_2_ | Unspecified (Method unclear) | Convulsions during loading dose injection: 1/22 (4.5%)  Note: one patient withdrew from the study due to convulsions during the loading dose phase and was not included in the final efficacy analysis (n = 20). | Not reported | PONV: No significant difference | PONV: No significant difference |
| Khan, 2019 | Standard monitoring | Unspecified (Monitored for objective signs like arrhythmia/convulsions, but no explicit mention of querying subjective symptoms) | 0/51 | 0/49 | 1. Respiratory depression: 0;  2. Nausea: 10/51 (19.6%);  3. Vomiting: 1/51 (2.0%);  4. Infection: 6/51 (11.8%);  5. Vasopressor required: 1/51 (2.0%). | 1. Respiratory depression: 1/49 (2.0%);  2. Nausea: 11/49 (22.4%);  3. Vomiting: 5/49 (10.2%);  4. Infection: 7/49 (14.3%);  5. Vasopressor required: 0. |
| Ghimire, 2020 | Standard monitoring + EtCO_2_ | Unspecified (Method unclear) | Intraoperative hypotension and bradycardia (requiring pharmacological intervention): 1/32 (3.1%) | Not reported | 1. Nausea: 5/32 (15.6%);  2. Vomiting: 2/32 (6.3%);  3. Antiemetic required: 3/32 (9.4%);  4. Sedation score > 2: 4/32 (12.5%). | 1. Nausea: 14/32 (43.8%);  2. Vomiting: 8/32(25%);  3. Antiemetic required: 11/32 (34.4%);  4. Sedation score > 2: 3/32 (9.4%). |
| Dai, 2020 | Standard monitoring + invasive arterial/central venous pressure measurement + BIS | Not clearly described (Relied on patient complaints) | No serious adverse events | No serious adverse events | 1. Somnolence 7/58 (12.1%);  2. Dry mouth 3/58 (5.2%). | 1. Dizziness 2/57(3.5%);  2. Somnolence 5/57(8.8%);  3. Nausea/Vomiting 1/57(1.8%). |
|  |  |  |  |  |  | (*continued on next page*) |
|  |  |  |  |  |  |  |

**Supplementary Table 4 (*continued*)**

| Study | Safety Monitoring Protocol | Active Assessment for LAST Symptoms | Adverse events possibly related to lidocaine (Lidocaine Group） | Adverse events possibly related to placebo (Control group) | Other Postoperative Adverse Events (Lidocaine Group) | Other Postoperative Adverse Events (Control Group) |
| --- | --- | --- | --- | --- | --- | --- |
| Toner, 2021 | Standard monitoring (intraop & PACU) + LOLIPOP local anaesthetic infusion monitoring tool | Yes (explicitly asked about systemic symptoms) | 1. Intraoperative hypotension requiring treatment: 45/74 (61%); intraoperative bradycardia requiring treatment: 7/74 (9%); dizziness, visual disturbance, metallic taste: 1/74 (1.4%).  2. Symptoms reported (PACU)**:** Tinnitus 3%; Dizziness 4%; Visual disturbances 3%; Metallic taste 7%.  3. Symptoms reported (Ward, 4-hrly): dizziness 5%; Visual disturbances 3%; Metallic taste 3%. | 1. Hypotension requiring treatment: 33/76(43%); intraoperative bradycardia requiring treatment: 4/76 (5%); sinus bradycardia: 1/76(1.3%); visual disturbances & metallic taste: 1/76(1.3%).  2. Symptoms reported (PACU): Tinnitus 1%, dizziness 3%; Visual disturbances 1%; metallic taste 5%.  3. Symptoms reported (Ward, 4-hrly): Paresthesia 4%; tinnitus 3%; dizziness 1%; metallic taste 3%. | 1. Subcutaneous catheter site events: 3/74 (4.1%);  2. Wound infection at 30 days: 5/74 (7%).  3. Medical Emergency Team activation (hypotension) and unplanned re-operation (surgical site haematoma): 1/74 (1.4%). | 1. Wound infection at 30 days: 2/76 (3%);  2. Medical Emergency Team activation (vasovagal episodes): 2/76 (2.6%); |
| Xia, 2022 | Standard monitoring + BIS | Not clearly described (Relied on patient complaints and routine observation by trained ward nurses) | No adverse or unintended effects were observed. | Not reported | None | None |
| Lu, 2022 | Standard monitoring + BIS (IBP only if necessary) | Unspecified (method not detailed) | No adverse or unintended effects were observed. | Not reported | Not reported | Not reported |
| Wallon, 2022 | Standard monitoring + EtCO2 + temperature monitoring | Unspecified (method not detailed) | Bradycardia: 1/57 (1.8%) | Ventricular premature contraction: 1/61(1.6%) | Not reported | Alcohol withdrawal tremor (1/61) |
|  |  |  |  |  |  | (*continued on next page*) |
|  |  |  |  |  |  |  |
|  |  |  |  |  |  |  |

**Supplementary Table 4 (*continued*)**

| Study | Safety Monitoring Protocol | Active Assessment for LAST Symptoms | Adverse events possibly related to lidocaine (Lidocaine Group） | Adverse events possibly related to placebo (Control group) | Other Postoperative Adverse Events (Lidocaine Group) | Other Postoperative Adverse Events (Control Group) |
| --- | --- | --- | --- | --- | --- | --- |
| Rekatsina, 2022 | Standard monitoring (implicit, intraoperative hemodynamics recorded) | Unspecified (evaluated, but method not stated) | Ventricular ectopic beats:1/25  Intraoperative hypotension & bradycardia more frequent vs. control (statistically non-significant) | Hypotension and bradycardia occurred less frequently than in study groups (statistically non-significant) | Not reported | Not reported |
| Hojski, 2024 | Routine monitoring (carefully recorded AEs) | Not clearly described (Relied on patient complaints) | Bradycardia, hypotension, metallic taste: 1/29 (3.4%) | Not reported | Not reported | Not reported |
| Abdel Rady, 2024 | Standard monitoring + EtCO2 + temperature monitoring | Unspecified (method not detailed) | Hypotension: 14/30 (46.7%), Bradycardia: 8/30 (26.7%). | Hypotension: 1/30 (3.3%); Bradycardia: 1/30 (3.3%). | Not reported | Not reported |

PONV: Postoperative Nausea and Vomiting. AE: Adverse Event. BIS: Bispectral Index. EtCO₂: End-tidal Carbon Dioxide. IBP: Invasive Blood Pressure. LAST: Local Anesthetic Systemic Toxicity. PACU: Post-Anesthesia Care Unit. PCA: Patient-Controlled Analgesia.

Denominators reflect the populations used for the respective safety outcomes and may differ from those used in the efficacy analyses.
