# Supplementary material for: Perioperative intravenous lidocaine for the prevention of chronic postsurgical pain: a systematic review and meta-analysis of randomized controlled trials
Source: Front Med (Lausanne). 2026 Sep 28;13:1927770. doi: 10.3389/fmed.2026.1927770 (PMC13654987; doi:10.3389/fmed.2026.1927770)
Supplement: Supplementary file 10 [file Table5.docx]

Supplementary Table 5

Descriptive summary of movement-evoked pain scores at 3 months.

| Study | Pain-evoking condition | Scale | Lidocaine,  n; mean ± SD | Control,  n; mean ± SD | MD (95% CI) |
| --- | --- | --- | --- | --- | --- |
| Khan 2019 | Ipsilateral arm abduction to 90° | NRS 0–10 | 51; 1.4 ± 1.4 | 49; 1.8 ± 1.3 | -0.40 (-0.94 to 0.14) |
| Abdel Rady 2024 | Movement | NRS 0–10 | 30; 1.3 ± 0.4 | 30; 2.3 ± 0.4 | -1.00 (-1.21 to -0.79) |
| Hojski 2024 | Coughing | VAS 0–10 | 28; 0.79 ± 1.71 | 24; 0.25 ± 0.82 | 0.54 (-0.20 to 1.28) |

MDs were calculated as lidocaine mean minus control mean using Welch’s two-sample t method.
No pooled analysis was undertaken because of differences in pain-evoking conditions and data sources.
